# Targeting hypoxia-inducible factor-1 in a hypoxidative stress model protects retinal pigment epithelium cells from cell death and metabolic dysregulation

**DOI:** 10.1038/s41420-025-02675-7

**Published:** 2025-08-14

**Authors:** Annika Schubert, Maria Eduarda Lobo Barbosa da Silva, Tabea Ambrock, Orbel Terosian, Anna Malyshkina, Claudia Padberg, Safa Larafa, Johann Matschke, Joachim Fandrey, Yoshiyuki Henning

**Affiliations:** 1 Institute of Physiology, University Hospital Essen, University of Duisburg-Essen, Essen, Germany; 2 Institute of Cell Biology (Cancer Research), University Hospital Essen, University of Duisburg-Essen, Essen, Germany; 3 German Cancer Consortium (DKTK) partner site Essen a partnership between DKFZ and University Hospital Essen, Essen, Germany

**Keywords:** Cell death, Molecular biology

## Abstract

Oxidative stress and hypoxia lead to dysfunction of retinal pigment epithelium (RPE) cells and are hallmarks of diseases such as age-related macular degeneration (AMD), the most common blinding disease in the elderly population. We have previously shown that a combination of these two risk factors, i.e. hypoxidative stress, exacerbates RPE cell death by ferroptosis. Hypoxia leads to stabilization of hypoxia-inducible factors (HIFs), key regulators of cellular adaptation to hypoxic conditions. In the present study, we have therefore investigated the roles of HIF-1 and HIF-2 in RPE cell death in a human RPE cell line under hypoxidative stress. For this purpose, we conducted siRNA-mediated knockdowns of the α-subunits of HIF-1 and HIF-2. We found that especially iron metabolism, in particular the expression of transferrin receptor 1 (TFR1) was affected by HIF-1α silencing, resulting in decreased intracellular iron levels and ferroptosis susceptibility. We also found that heme oxygenase 1 (HO-1) contributed to cell death by hypoxidative stress. In addition, we also observed that cell metabolism was improved by HIF-1α silencing under hypoxia, most likely contributing to the protective effect. Furthermore, we identified an FDA-approved small molecule inhibitor, Vorinostat, to downregulate HIF-1α, TFR1, and HO-1 and improve cell metabolism, which eventually resulted in a full rescue of RPE cells from hypoxidative stress-induced cell death. In conclusion, this study highlights the importance of considering targeted HIF inhibition as a promising approach to protect RPE cells from degeneration.

## Introduction

The retinal pigment epithelium (RPE), a monolayer of pigmented cells located adjacent to the photoreceptors, serves as a gatekeeper regulating the transport of nutrients, oxygen, and ions from the choriocapillaris to the photoreceptors as well as the disposal of metabolic waste. In addition, RPE cells protect photoreceptors from light-induced damage, support the renewal of photoreceptor outer segments by phagocytosis, and execute the visual cycle [1]. Consequently, dysfunction and degeneration of RPE cells lead to photoreceptor degeneration [2] and diseases such as age-related macular degeneration (AMD), the leading cause of vision loss in the elderly population. Various cell death mechanisms might be involved in RPE degeneration [3]. Currently, ferroptosis is gaining attention as the primary mechanism leading to RPE cell atrophy in vitro and in vivo [4–8]. Ferroptosis is a non-apoptotic mode of cell death linked to lipid peroxidation of polyunsaturated fatty acids. Lipid peroxidation is driven by hydroxyl radicals, which are produced in the Fenton reaction with intracellular labile iron and H_2_O_2_ serving as substrates [9]. As such, RPE cell death both in vitro and in vivo decreased with reducing intracellular iron levels [4, 7, 10]. In addition, elevated iron levels in the retina, RPE and surrounding tissue were found in AMD patients, further supporting their clinical relevance [11–14].

The retina and RPE cells have high energy and oxygen demands, and combined with significant light exposure, this promotes the formation of reactive oxygen species (ROS) [2]. Furthermore, there is increasing evidence that hypoxia promotes RPE dysfunction with age, because age-related structural changes in the blood-retinal-barrier, including thickening of the Bruch’s membrane, deposits of metabolic waste products (i.e. drusen), and reduced choroidal blood flow impair oxygen supply and thus energy metabolism of RPE cells and photoreceptors [15–19]. Hypoxia leads to the accumulation of hypoxia-inducible factors (HIFs), dimeric transcription factors composed of an oxygen-labile α-subunit and a constitutively expressed β-subunit located in the nucleus [20]. Under physiological oxygen levels, α-subunits are constantly degraded by the proteasome following hydroxylation by oxygen-dependent prolyl hydroxylases 1-3 (PHD1, PHD2, and PHD3) [21]. Under hypoxia, degradation of HIF α-subunits is inhibited, resulting in the dimerization of the α- and the β-subunit. The two major HIF isoforms HIF-1 and HIF-2 regulate a cascade of hypoxia-induced adaptations to ensure the survival of a cell under hypoxia. These adaptations include promoting neovascularization and shifting metabolism from aerobic to anaerobic pathways. Albeit being protective in the first place, chronic activation of the HIF pathway plays a key role in retinal pathologies [18, 19, 22].

To the best of our knowledge, systematic investigation of the interplay between HIF signaling and oxidative stress on RPE cells is lacking, although it is likely to assume that the interplay between oxidative stress and hypoxia creates a microenvironment that fosters RPE dysfunction. To address this knowledge gap, we have previously established a combination model of HIF stabilization and oxidative stress, i.e., a hypoxidative stress model, using a human RPE cell line (ARPE-19) [4]. For this purpose, we induced oxidative stress by sodium iodate (NaIO_3_) and constantly activated HIF signaling with the pharmacological PHD inhibitor dimethyloxalylglycine (DMOG). We found that the combination of HIF stabilization and NaIO_3_ treatment exacerbated oxidative stress-induced cell death in ARPE-19 cells. Ferroptosis was identified as the primary cell death mechanism and iron metabolism was especially altered by hypoxidative stress [4].

In the present study, we induced hypoxidative stress in ARPE-19 cells by treating the cells with NaIO_3_ under hypoxic conditions. In this model, we aimed at identifying the differential roles of HIF-1 and HIF-2 in ferroptosis aggravation. Furthermore, we tested effects on cell metabolism and validated different commercially available drugs for their potential to protect RPE cells from cell death and metabolic dysregulation.

## Results

### HIF-1 aggravates ferroptotic cell death by hypoxidative stress

**Fig. 1:**
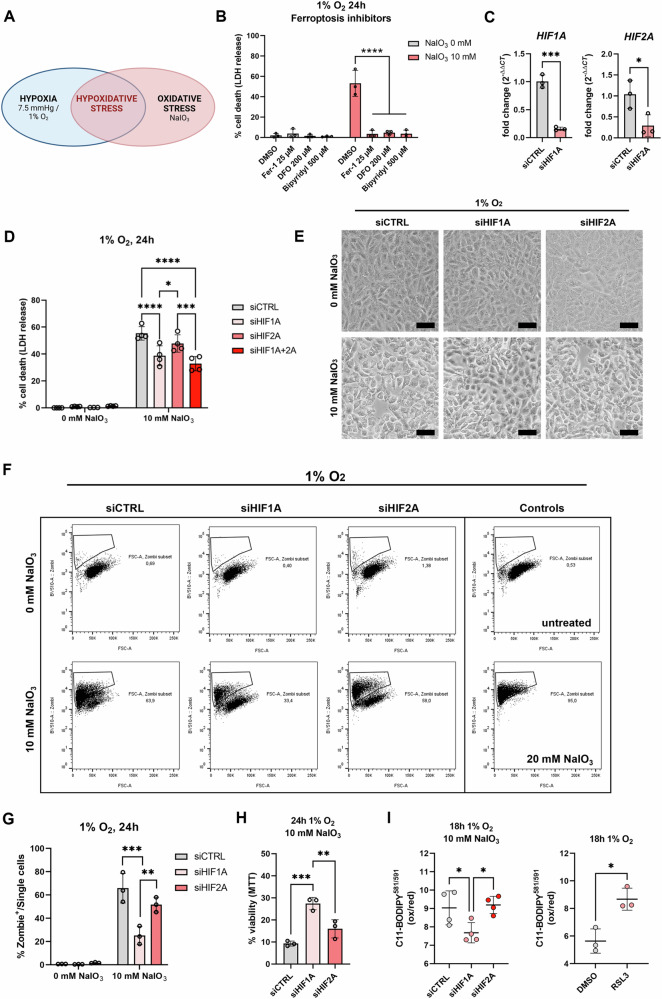
Effects of HIF-1α and HIF-2α knockdown on hypoxidative stress-induced cell death in ARPE-19 cells. **A** Hypoxidative stress is induced by the combination of hypoxia treatment in a hypoxia chamber at 1% O_2_ (7.5 mmHg O_2_) and oxidative stress induced by NaIO_3_. Created in BioRender.com. **B** Hypoxidative stress resulted in substantial cell death. Ferroptosis inhibitors protected cells from hypoxidative stress-induced cell death (*N* = 3). **C** Knockdown efficiency of siRNA-mediated silencing of HIF-1α (left panel) and HIF-2α (*N* = 3). **D** HIF-1α knockdown protected ARPE-19 cells from hypoxidative stress-induced cell death (*N* = 4). **E** Micrographs of knockdown cells treated either under hypoxia alone or hypoxidative stress conditions showed many regions with intact cells in the HIF-1α knockdown group. **F** Representative plots of flow cytometry analyses using Zombie Aqua dye to quantify cell death. **G** Analysis of Zombie Aqua-positive cells confirmed that cell death was significantly reduced by HIF-1α knockdown (*N* = 3). **H** The protective effect of HIF-1α knockdown was further confirmed by MTT assay (*N* = 3). **I** C11-BODIPY^581/591^ staining revealed that lipid peroxidation is downregulated by HIF-1α knockdown pointing towards ferroptosis inhibition (left panel, *N* = 4). RSL3, a ferroptosis inducer, was used in parallel as positive control (right panel, *N* = 3). LDH, flow cytometry and MTT assays were statistically analyzed with two-way ANOVA followed by Tukey’s multiple comparisons test, knockdown efficiencies were analyzed with *t* test, and lipid peroxidation assays were analyzed with one-way ANOVA followed by Tukey’s multiple comparison’s test. All data are expressed as mean ± SD. *p < 0.05, **p < 0.01, ***p < 0.001, and ****p < 0.0001. Scale bar = 50 μm.

We treated ARPE-19 cells under hypoxic (1% O_2_) or hypoxidative stress (1% O_2_ and 10 mM NaIO_3_) conditions (Fig. 1A) to reproduce previous findings where hypoxidative stress was induced by the PHD inhibitor DMOG and NaIO_3_ [4]. As in the previous hypoxidative stress model, cell death induced by NaIO_3_ under hypoxia was mainly driven by ferroptosis, as evidenced by the complete rescue of cells from cell death by co-treatment with the lipid peroxidation inhibitor ferrostatin-1 (Fer-1), and two iron chelators (deferoxamine mesylate [DFO]; bipyridyl), known to inhibit ferroptosis (Fig. 1B). As hypoxia treatment induces stabilization of both, HIF-1α and HIF-2α, we conducted siRNA-mediated knockdowns of HIF-1α and HIF-2α, which resulted in significant silencing of both targets (Fig. 1C). Using this knockdown model, we assessed cell death by different assays (Fig. 1D–H). Quantification of cell death by LDH release, demonstrated that HIF-1α silencing resulted in a significant reduction of cell death by approximately 20%, while HIF-2α silencing had no significant effect on cell death (Fig. 1D). Microscopic assessment of cell morphology revealed that HIF-1α knockdown cells retained many patches of intact cells, while the other groups showed homogenous cell death (Fig. 1E). Using Zombie Aqua, a fixable viability dye for flow cytometry (Fig. 1F, G), and MTT assay (Fig. 1H) we confirmed that HIF-1α knockdown protects ARPE-19 cells from cell death. To assess whether ferroptosis inhibition is the primary mechanism responsible for the reduction in cell death observed following HIF-1α knockdown, we quantified lipid peroxidation, a hallmark of ferroptosis, using C11-BODIPY 581/591 staining. Our findings revealed a significant decrease in lipid peroxidation in HIF-1α knockdown cells (Fig. 1I, left). As a positive control for lipid peroxidation, the ferroptosis inducer RSL3 was used (Fig. 1I, right).

### HIF-1α knockdown reduces intracellular iron levels

**Fig. 2:**
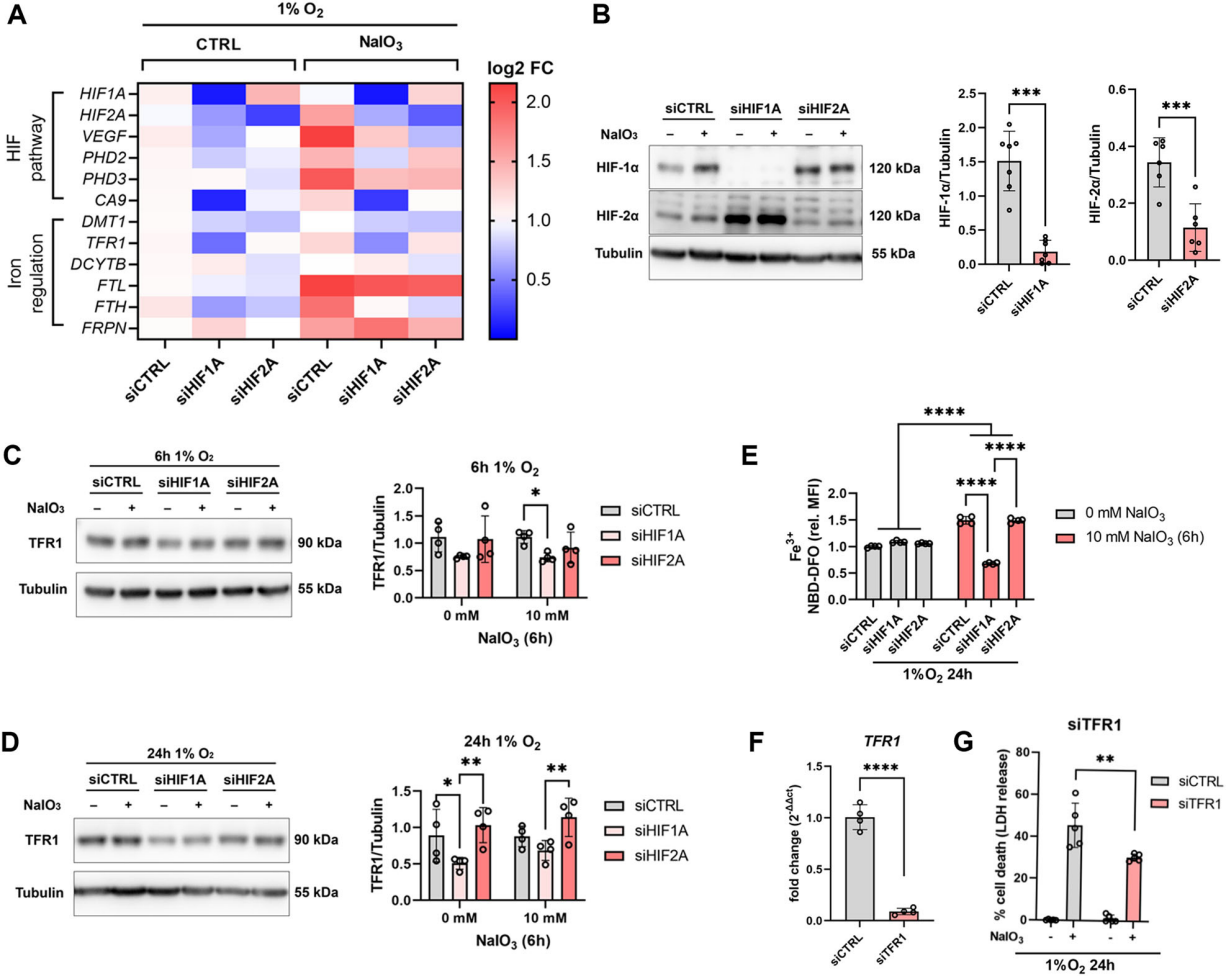
Effects of HIF knockdown on iron regulation under hypoxia and hypoxidative stress. **A** Expression of HIF-associated and iron regulatory genes in ARPE-19 cells treated under hypoxia or hypoxidative stress conditions (*N* = 4–6). **B** Representative images of Western blots of HIF-1α, HIF-2α, and Tubulin as loading control and quantification of HIF-1α and HIF-2α protein levels (*N* = 6–7) in ARPE-19 cells with HIF-1α and HIF-2α knockdown treated under hypoxia or hypoxidative stress conditions for 24 h. **C**, **D** Representative images and quantification of TFR1 protein levels normalized to Tubulin revealed that HIF-1α knockdown resulted in a significant downregulation of TFR1 after (**C**) 6 h and (**D**) 24 h (24 h treatments consisted of 18 h preincubation at 1% O_2_ followed by 6 h incubation with 10 mM NaIO_3_ at 1% O_2_ to avoid cell death by NaIO_3_) (*N* = 4). **E** Quantification of intracellular Fe^3+^ by flow cytometry in ARPE-19 cells with HIF-1α and HIF-2α knockdown showed that intracellular Fe^3+^ was downregulated by HIF-1α knockdown under hypoxidative stress conditions. **F** Knockdown efficiency of siRNA-mediated knockdown of TFR1 quantified by qRT-PCR. **G** TFR1 knockdown under hypoxidative stress conditions protected cells from cell death quantified by LDH assay. LDH assays were statistically analyzed with two-way ANOVA followed by Tukey’s multiple comparisons test, Western blot data were statistically analyzed with mixed effects model followed by Šídák’s multiple comparisons test, and knockdown efficiencies were analyzed with *t* test. All data are expressed as mean ± SD. *p < 0.05, **p < 0.01, ***p < 0.001, and ****p < 0.0001.

Intracellular iron is a critical determinant for ferroptosis susceptibility. Therefore, we analyzed the influence of hypoxia and hypoxidative stress on the expression of HIF-associated genes and iron regulators in HIF-1α and HIF-2α knockdown cells (Fig. 2A). Both, HIF-1α and HIF-2α knockdown resulted in a significant downregulation of *HIF1A* and *HIF2A* gene expression, respectively (Fig. 2A; Supplementary Fig. S1). In line, *CA9* (coding for carbonic anhydrase 9), a HIF-1 target gene, was significantly downregulated by HIF-1α knockdown and *VEGF* (coding for the vascular endothelial growth factor), another HIF target gene, was downregulated by HIF-2α knockdown (Fig. 2A; Supplementary Fig. S1). Contrary to our expectations, the HIF target genes *PHD2*, *PHD3*, (Fig. 2A; Supplementary Fig. S1) and *ADM* (coding for adrenomedullin; Supplementary Fig. S1) were not differentially regulated by HIF knockdown, which might require longer incubation times. Still, we found a significant upregulation of *PHD3* expression by hypoxidative stress (Fig. 2A; Supplementary Fig. S1). Among the genes coding for iron regulatory proteins (Fig. 2A; Supplementary Fig. S2), we found especially *TFR1*, coding for the iron importer transferrin receptor 1, to be downregulated by HIF-1α knockdown (Fig. 2A; Supplementary Fig. S2). *FTL*, coding for the light chain of ferritin, an intracellular iron storage protein, and *FRPN*, coding for the iron exporting protein ferroportin, were significantly upregulated by hypoxidative stress treatment (Fig. 2A; Supplementary Fig. S2). Assessment of HIF knockdown via Western blot confirmed successful silencing of HIF-1α and HIF-2α (Fig. 2B), which was in line with gene expression data (Fig. 1C). Based on gene expression analyses, we took a closer look at the iron importer TFR1 in HIF knockdown cells by Western blot (Fig. 2C, D) after 6 h and 24 h of hypoxia treatment. To avoid cytotoxicity by hypoxidative stress in samples treated for 24 h, we preincubated the cells for 18 h at 1% O_2_ followed by treatment with 10 mM NaIO_3_ for 6 h at 1% O_2_. Analysis of TFR1 protein levels by Western blot revealed that TFR1 is downregulated in HIF-1α knockdown cells after 6 h and 24 h of hypoxia and hypoxidative stress treatment (Fig. 2C, D). This expression pattern suggests that HIF-1α knockdown may contribute to the downregulation of intracellular iron via downregulation of TFR1. Therefore, we analyzed intracellular iron levels by flow cytometry in HIF knockdown cells. We found that hypoxidative stress conditions resulted in elevated levels of intracellular iron compared to hypoxic cells. As expected, HIF-1α knockdown resulted in a significant downregulation of intracellular iron (Fig. 2E). To provide evidence for the causal relationship between TFR1, intracellular iron, and cell death, we conducted siRNA-mediated knockdown of TFR1 (Fig. 2F). Analysis of cell death supported a causal relationship, as ARPE-19 cells were protected from cell death by TFR1 knockdown (Fig. 2G).

### HIF-1α knockdown enhances NRF2 signaling, and HO-1 induction promotes cell death upon hypoxidative stress

**Fig. 3:**
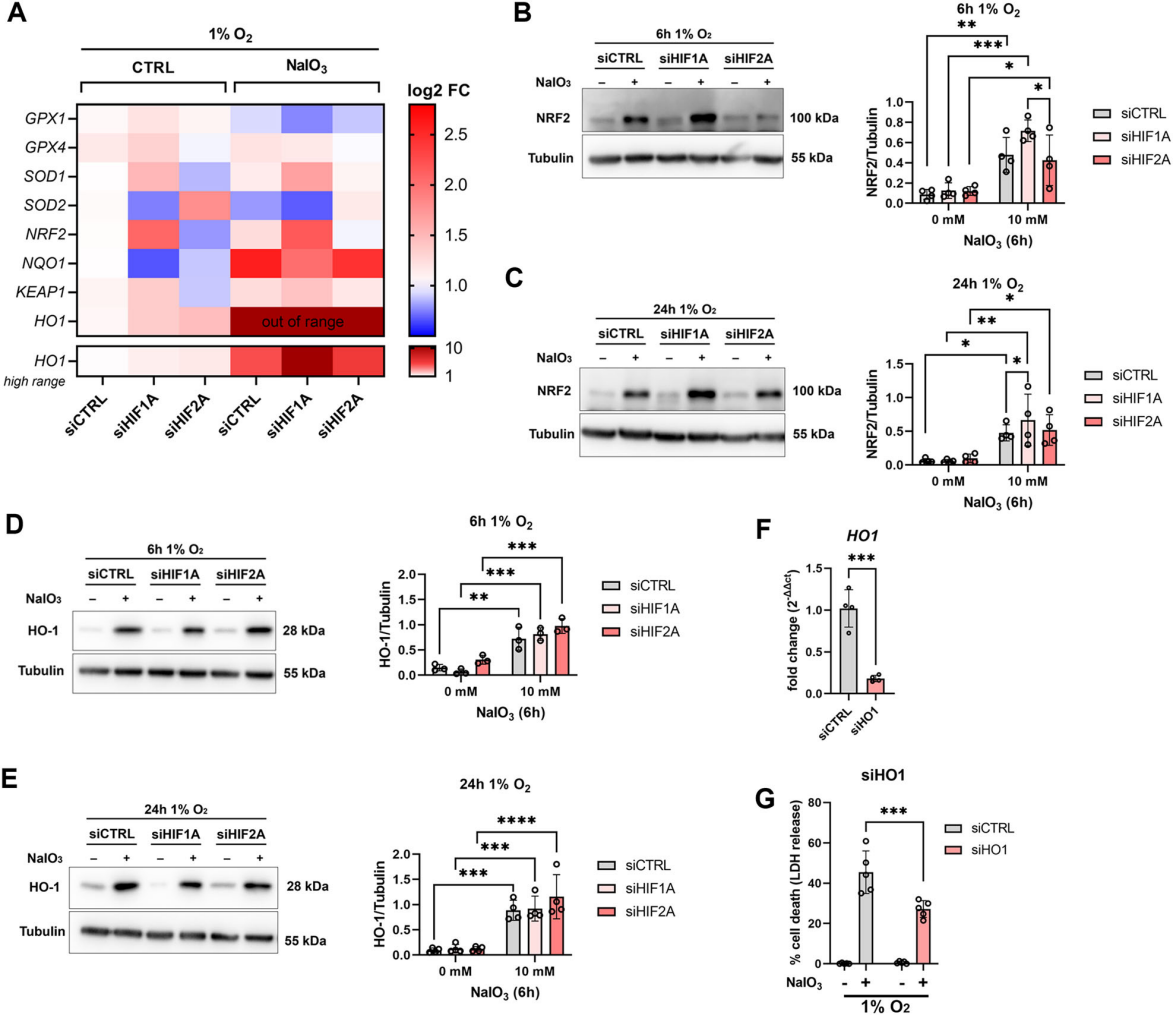
Effects of HIF knockdown on regulators of the antioxidative response under hypoxia and hypoxidative stress. **A** Expression of antioxidative response genes in ARPE-19 cells treated under hypoxia or hypoxidative stress conditions (*N* = 5–6). The fold change in *HO1* expression under hypoxidative stress conditions exceeded the range observed for the other genes. Consequently, *HO1* is presented separately using a distinct, higher range. **B**, **C** Representative images and quantification of Western blots of NRF2 and Tubulin as loading control from ARPE-19 cells with HIF-1α and HIF-2α knockdown treated under hypoxia or hypoxidative stress conditions for (**B**) 6 h and (**C**) 24 h (24 h treatments consisted of 18 h preincubation at 1% O_2_ followed by 6 h incubation with 10 mM NaIO_3_ at 1% O_2_ to avoid cell death by NaIO_3_.). The analysis revealed that NRF2 was significantly upregulated by HIF-1α knockdown under hypoxidative stress (*N* = 4). **D**, **E** Representative images and quantification of Western blots of HO-1 and Tubulin as loading control from ARPE-19 cells with HIF-1α and HIF-2α knockdown treated under hypoxia or hypoxidative stress conditions for (**D**) 6 h and (**E**) 24 h (24 h treatments consisted of 18 h preincubation at 1% O_2_ followed by 6 h incubation with 10 mM NaIO_3_ at 1% O_2_ to avoid cell death by NaIO_3_). The analysis revealed that HO-1 was significantly upregulated by hypoxidative stress (*N* = 3–4). **F** Knockdown efficiency of siRNA-mediated knockdown of HO-1 quantified by qRT-PCR (*N* = 4). **G** Quantification of cell death by LDH assay showed that HO-1 knockdown protected ARPE-19 cells from hypoxidative stress-induced cell death (*N* = 5). LDH assays were statistically analyzed with two-way ANOVA followed by Tukey’s multiple comparisons test, Western blot data were statistically analyzed with mixed-effect model followed by Šídák’s multiple comparisons test, and knockdown efficiencies were analyzed with *t* test. All data are expressed as mean ± SD. ^*^p < 0.05, ^**^p < 0.01, ^***^p < 0.001, and ^****^p < 0.0001.

Among the antioxidative response genes especially *SOD1* (coding for superoxide dismutase 1), *SOD2* (coding for superoxide dismutase 2), *NRF2* (coding for nuclear factor erythroid 2-related factor 2), *HO1* (coding for heme oxygenase 1), and *NQO1* (coding for NAD(P)H:quinone oxidoreductase 1) were differentially regulated by HIF knockdown and/or hypoxidative stress (Fig. 3A; Supplementary Fig. S3). HIF-1α knockdown had the most pronounced effects on *NRF2* and *HO1* expression. In addition, expression of *NQO1*, an NRF2 target gene, was significantly upregulated by hypoxidative stress (Fig. 3A). Based on gene expression data, we selected NRF2 and HO-1 for analysis of protein levels by Western blot (Fig. 3B–E). We again analyzed cells incubated for 6 h and 24 h (18 h preincubation at 1% O_2_ followed by 6 h NaIO_3_ treatment at 1% O_2_) at 1% O_2_ either with our without 10 mM NaIO_3_. As expected, hypoxidative stress significantly upregulated NRF2 protein levels at both timepoints. Moreover, NRF2 levels were significantly higher in HIF-1α knockdown cells under hypoxidative stress (Fig. 3B, C). HO-1 protein levels were not differentially regulated by HIF silencing but hypoxidative stress resulted in a significant upregulation of HO-1 compared to hypoxic cells at both timepoints (Fig. 3D, E). To assess a potential functional link between HO-1 and cell death, we conducted an siRNA-mediated knockdown of HO1 (Fig. 3F). HO-1 silencing protected ARPE-19 cells from cell death, suggesting that HO-1 exhibits a cytotoxic role under hypoxidative stress conditions (Fig. 3G).

### HIF-1α knockdown improves mitochondrial respiration and glycolytic capacity under hypoxia and hypoxidative stress

**Fig. 4:**
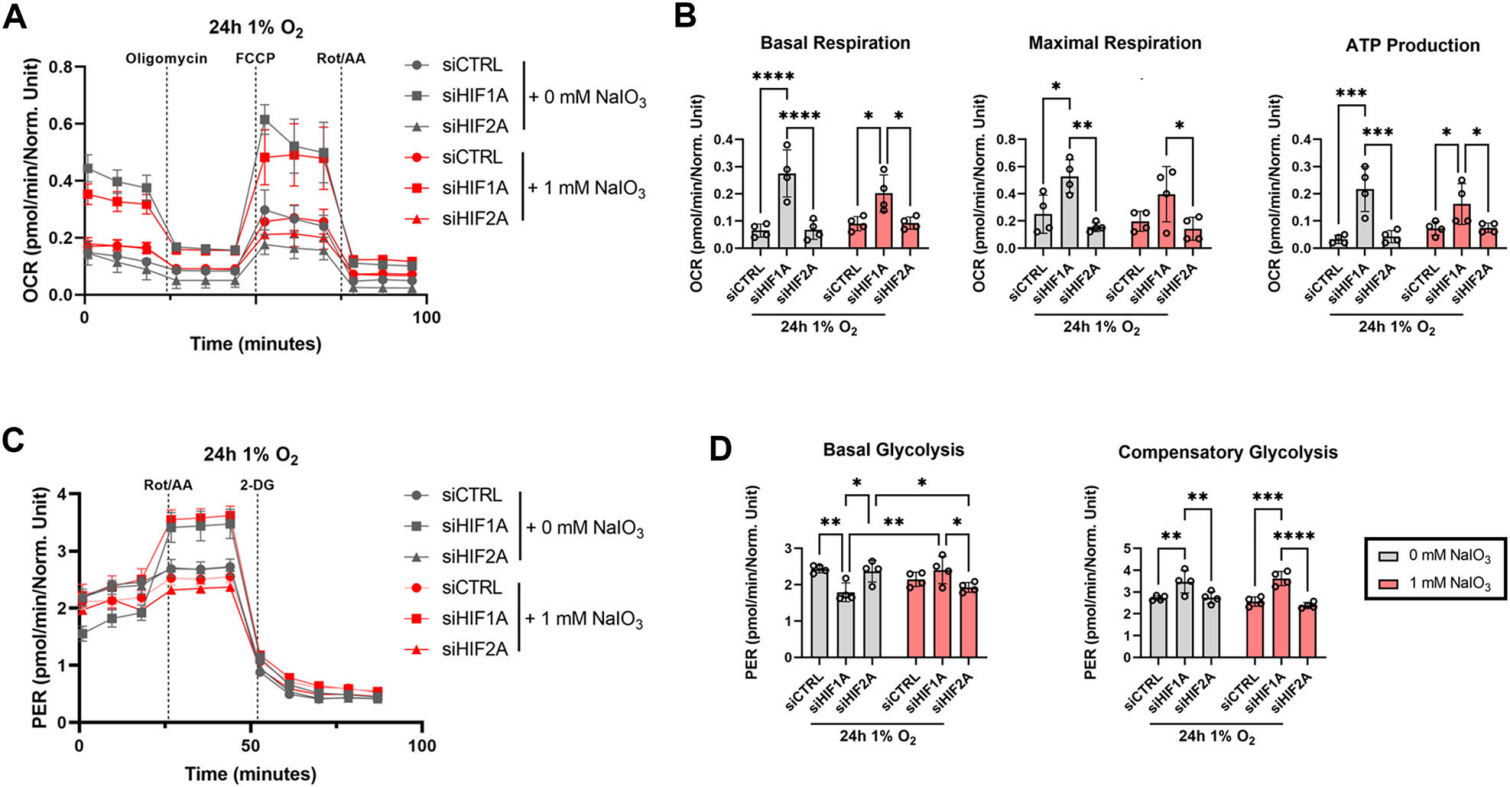
Metabolic flux analysis of ARPE-19 cells. ARPE-19 cells with HIF-1α and HIF-2α knockdown were treated under hypoxia or hypoxidative stress conditions for 24 h. **A** A Mitochondrial Stress Test was performed in order to assess mitochondrial function. Mean oxygen consumption rates (OCR) normalized to Hoechst staining are shown (*N* = 4). **B** Key parameters of mitochondrial function (basal respiration, maximal respiration, ATP production) were significantly improved by HIF-1α knockdown (*N* = 4). **C** A Glycolytic Rate Assay was performed in order to assess glycolytic flux. Mean proton efflux rates (PER) normalized to Hoechst staining are shown (*N* = 4). **D** Basal glycolysis was downregulated in HIF-1α knockdown cells under hypoxia and downregulated in HIF-2α knockdown cells under hypoxidative stress conditions (*N* = 4). Compensatory glycolysis was significantly upregulated in HIF-1α knockdown cells (*N* = 4). Data were statistically analyzed with two-way ANOVA followed by Tukey’s multiple comparisons test. Data are expressed as mean ± SEM (**A**, **C**) or mean ± SD (**B**, **D**). ^*^p < 0.05, ^**^p < 0.01, ^***^p < 0.001, and ^****^p < 0.0001.

Metabolism plays an important role in oxidative stress response. Here, we conducted a mitochondrial stress test and a glycolytic rate assay using a Seahorse Bioanalyzer to test whether HIF-1α and/or HIF-2α knockdown can enhance cellular metabolism under hypoxic and hypoxidative stress conditions (Fig. 4). The mitochondrial stress test revealed that important measures of mitochondrial function (basal respiration, maximal respiration, and mitochondria-related ATP production) were upregulated by HIF-1α knockdown (Fig. 4A, B). Furthermore, HIF-1α knockdown significantly improved compensatory glycolysis, while basal glycolysis was less affected, albeit a drop in HIF-1α knockdown cells under hypoxia (Fig. 4C, D), which might be explained by higher mitochondrial respiration (Fig. 4B).

### Vorinostat and SP2509 fully rescued ARPE-19 cells from hypoxidative stress-induced cell death

**Fig. 5:**
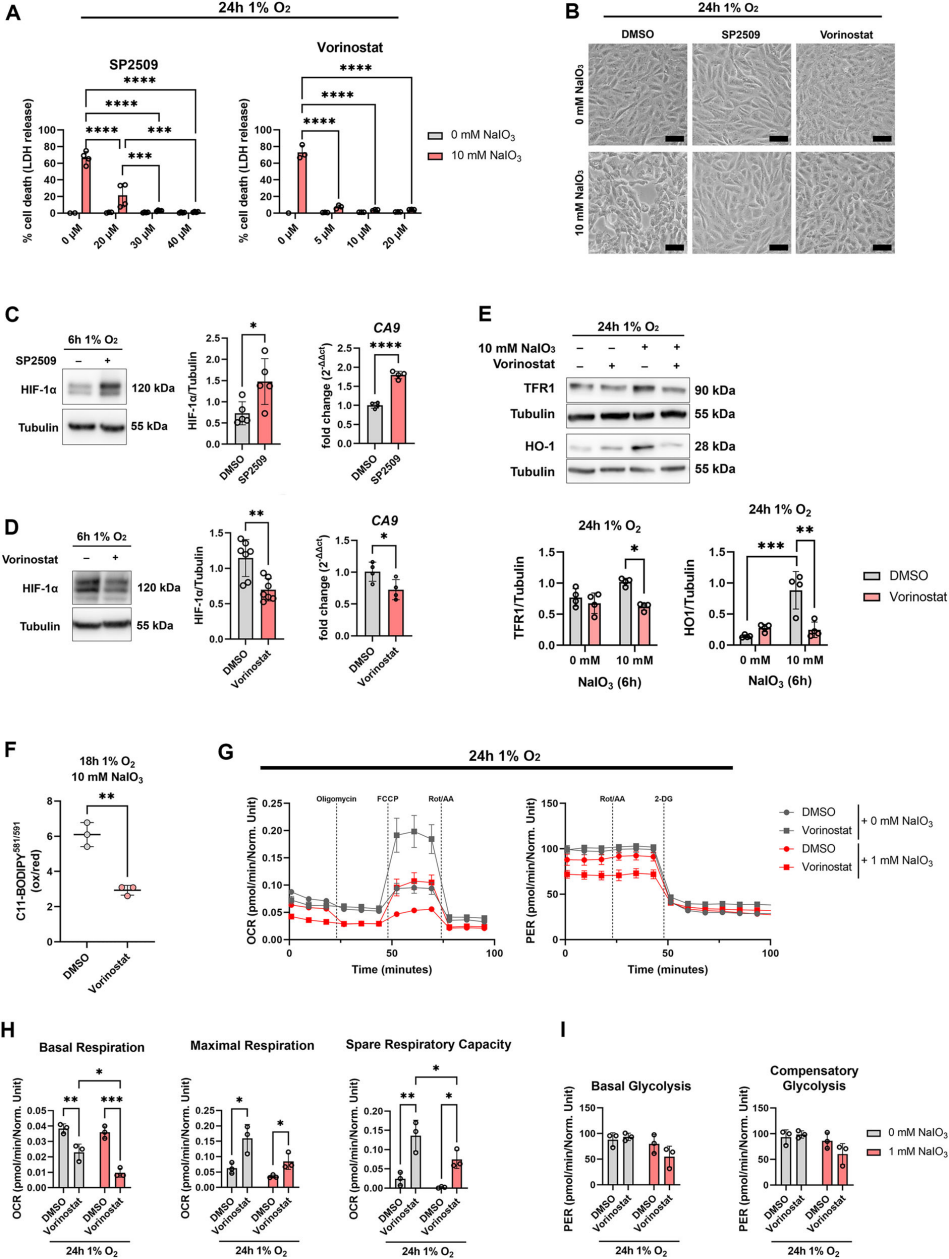
Effects of SP2509 and Vorinostat on ARPE-19 cells. **A** ARPE-19 cells were treated under hypoxia or hypoxidative stress conditions for 24 h with SP2509, Vorinostat, or vehicle (DMSO) and cell death was determined with LDH assays (*N* = 3–4). **B** Micrographs of ARPE-19 cells treated with 30 µM SP2509 and 10 µM Vorinostat confirmed that both inhibitors fully rescued cells from cell death. **C**, **D** Western blots of ARPE-19 cells treated under hypoxia for 6 h revealed that 30 µM SP2509 increased and 10 µM Vorinostat significantly decreased HIF-1α protein levels (*N* = 5–7). Consequently, the HIF-1 target gene *CA9* was upregulated by SP2509 and downregulated by Vorinostat. **E** ARPE-19 cells treated with 10 µM Vorinostat for 24 h under hypoxidative stress exhibited decreased TFR1 and HO-1 protein levels. **F** ARPE-19 cells were treated under hypoxidative stress conditions for 18 h together with 10 µM Vorinostat or vehicle (DMSO). C11-BODIPY^581/591^ treatment revealed that lipid peroxidation was significantly decreased by Vorinostat (*N* = 3). **G** To test metabolic effects of Vorinostat on metabolism of stressed ARPE-19 cells, cells were treated under hypoxia or hypoxidative stress conditions with 10 µM Vorinostat or vehicle (DMSO) for 24 h and metabolic flux analyses were conducted using a Seahorse Bioanalyzer. A Mitochondrial Stress Test (left) and a Glycolytic Rate Assay (right) were performed to assess mitochondrial function and glycolytic flux. **H** While basal respiration was significantly decreased by Vorinostat treatment, maximal respiration and spare respiratory capacity were significantly increased by Vorinostat. **I** Glycolytic Flux was not affected by the treatments. LDH and Seahorse assays were statistically analyzed with two-way ANOVA followed by Tukey’s multiple comparisons test, Western blots were analyzed with *t* test (**C**, **D**) or mixed-effects model followed by Šídák’s multiple comparisons test (**E**), and lipid peroxidation data were analyzed with *t* test. Data are expressed as mean ± SD (except for G: mean ± SEM). ^*^p < 0.05, ^**^p < 0.01, ^***^p < 0.001, and ^****^p < 0.0001. Scale bar: 50 µm.

We tested a panel of small molecule inhibitors with a potential inhibitory effect on HIF signaling to identify ways for pharmacological protection from hypoxidative stress-induced cell death (Supplementary Fig. S4). We treated ARPE-19 cells with each inhibitor under hypoxidative stress conditions and measured cell death using an LDH assay. We found that especially SP2509 and Vorinostat fully rescued ARPE-19 cells from cell death without having adverse effects (Fig. 5A). Therefore, for further analyses, we focused on SP2509, an inhibitor of lysine-specific demethylase 1 [23], and Vorinostat (SAHA), an FDA-approved histone deacetylase inhibitor [24]. Micrographs of treated cells additionally confirmed that SP2509 and Vorinostat fully protected cells from hypoxidative stress-induced cell death (Fig. 5B). We further validated whether both inhibitors downregulate HIF-1α on protein level. Surprisingly, SP2509 increased HIF-1α protein levels, while Vorinostat treatment resulted in a downregulation of HIF-1α protein expression under hypoxia (Fig. 5C, D). Gene expression of *CA9*, a HIF-1 target gene, further confirmed that SP2509 increased and Vorinostat decreased HIF-1 transcriptional activity (Fig. 5C, D). Given the inhibitory effects of Vorinostat on HIF-1 signaling, subsequent analyses were performed using Vorinostat.

### Vorinostat treatment protected ARPE-19 cells from lipid peroxidation and improved mitochondrial respiration

Similar to the effects observed by HIF-1α silencing, Vorinostat significantly decreased TFR1 protein levels under hypoxidative stress after 24 h (Fig. 5E). Given the involvement of HO-1 in hypoxidative stress-induced cell death, we also analyzed HO-1 protein levels. The analysis revealed that Vorinostat treatment under hypoxidative stress resulted in a significant downregulation of HO-1 to control levels (Fig. 5E). Next, we aimed to ascertain whether the protective effect of Vorinostat is based on the ability to overcome a critical step in the ferroptosis induction process, which we have previously demonstrated occurs via lipid peroxidation in cells where HIF-1α has been knocked down (Fig. 1I). For this purpose, we measured lipid peroxidation by C11-BODIPY staining and found that Vorinostat significantly decreased lipid peroxidation levels in ARPE-19 cells treated under hypoxidative stress conditions (Fig. 5F). Next, we analyzed whether mitochondrial metabolism is enhanced by Vorinostat treatment (Fig. 5G–I), as observed in HIF-1α knockdown cells (Fig. 4A, B). While basal respiration was significantly reduced by Vorinostat in cells treated under hypoxia and hypoxidative stress conditions, maximum respiration and spare respiratory capacity, a measure of the ability to respond to increased energy demand and/or under stress, were significantly increased by Vorinostat treatment (Fig. 5G, H). In contrast, glycolytic capacity was not significantly affected by Vorinostat treatment (Fig. 5G, I).

## Discussion

We have previously described that oxidative stress in combination with pan-HIF stabilization using DMOG potentiates oxidative stress and RPE degeneration by ferroptosis [4]. In the present study, we established a hypoxidative stress model using a human RPE cell line to selectively investigate how HIF-1 and HIF-2 affect RPE integrity and metabolism. We could show that hypoxidative stress induced by NaIO_3_ under hypoxic conditions induced ferroptosis in ARPE-19 cells, which is in line with our previous study using DMOG instead of hypoxia [4]. Furthermore, we could provide three lines of evidence that silencing of HIF-1α protects RPE cells from cell death induced by hypoxidative stress: (i) alterations in iron metabolism, (ii) antioxidative mechanisms, and (iii) improvements in energy metabolism.

First, we found that a prerequisite of ferroptosis, intracellular iron, was downregulated by HIF-1α knockdown, most likely because of downregulated iron import. In particular, analysis of gene expression and protein levels revealed that, in consequence of HIF-1α silencing, TFR1 was downregulated. TFR1 was already reported as a HIF-1 target gene [25] and in a previous study, we have also observed the induction of TFR1 expression by treatment with DMOG and NaIO_3_ [4]. Furthermore, TFR1 silencing under hypoxidative stress conditions showed a protective effect to a similar extent as HIF-1α silencing. This strongly suggests that upregulation of TFR1 by HIF-1 under hypoxidative stress conditions promotes iron uptake and increases ferroptosis susceptibility. However, in contrast to our findings, HIF-1 was reported to be protective against ferroptosis in many cases [26–31]. However, HIF-1 and HIF-2 also promote ferritinophagy in the liver by NCOA4 expression to mobilize iron after blood loss [32]. Although ferritin was upregulated by hypoxidative stress in our previous study [4] and not influenced by HIF knockdown (not shown), these contradicting findings suggest that the role of HIF-1 and HIF-2 in the regulation of ferroptosis is context- and cell type-dependent and cannot be generalized. Furthermore, HIF-1 and HIF-2, in particular, have distinct yet overlapping functions in regulating iron homeostasis: While HIF-1 regulates genes involved in iron uptake and storage, such as TFR1 and HO-1 [25, 33], HIF-2 directly regulates the transcription of genes essential for iron absorption, such as divalent metal transporter 1 (DMT1) and ferroportin. Mice lacking intestinal HIF-2α exhibit impaired iron absorption and systemic iron deficiency, underscoring its vital role in maintaining iron balance [34, 35]. Consequently, our findings highlight the role of HIF-1 in cellular iron uptake and suggest that HIF-1, rather than HIF-2, is a more promising therapeutic target for reducing ferroptosis susceptibility.

Second, NRF2, a key regulator of antioxidative mechanisms, was upregulated in HIF-1α but not HIF-2α knockdown cells. As expected, we also observed a significant upregulation of NRF2 by NaIO_3_ in all groups, along with an upregulation of its target genes *NQO1* and *HO1*. The NRF2 response to oxidative stress was shown to decline with age in the RPE of mice and has been implicated with dysregulation of RPE metabolism and AMD pathophysiology [2, 36]. HO-1 was also upregulated by HIF-1α silencing under hypoxidative stress, but only on gene expression level. On the protein level, HO-1 was upregulated in all hypoxidative stress groups. HO-1 has a protective but also a prooxidative role depending on the context, as it catalyzes heme degradation, leading to the release of ferrous iron [37]. By HO-1 knockdown, we could confirm that HO-1 in the context of hypoxidative stress has a prooxidative role, which is consistent with previous findings, where HO-1 was shown to exacerbate RPE ferroptosis [7].

Third, we could demonstrate that HIF-1α silencing improves energy metabolism of RPE cells under hypoxia and hypoxidative stress. HIF signaling under hypoxia is a prerequisite for metabolic adaptations to hypoxia, resulting in a metabolic shift from mitochondrial respiration to anaerobic glycolysis [38]. However, glycolysis is less efficient in ATP production compared to mitochondrial respiration [39]. Furthermore, a switch to glycolysis might compromise antioxidative defense mechanisms required to combat oxidative stress [40]. Since age-related structural changes in the blood-retinal-barrier can promote hypoxia in RPE cells and the retina [15–19], targeted inhibition of the HIF pathway is a promising approach to improve mitochondrial function as an early intervention strategy against age-related diseases such as AMD [41].

As a pharmacological approach, we identified Vorinostat, an FDA-approved histone deacetylase inhibitor for the treatment of T-cell lymphoma, as a promising agent to protect RPE cells from hypoxidative stress-induced cell death. Vorinostat treatment fully prevented cell death, reduced lipid peroxidation and enhanced cellular metabolism in ARPE-19 cells. Moreover, Vorinostat treatment decreased TFR1 protein levels, suggesting that reduced iron uptake may contribute to the protective effect of Vorinostat. Western blot analysis confirmed the ability of Vorinostat to downregulate HIF-1α protein levels. In line, *CA9* expression, a HIF-1 target gene, was downregulated by Vorinostat. These effects on HIF-1 have also been demonstrated previously [24, 42]. Nonetheless, the protective effect of Vorinostat was significantly greater than that of HIF-1α silencing via siRNA. While Vorinostat fully rescued ARPE-19 cells from cell death, HIF-1α silencing only reduced cell death by approximately 20%. These findings suggest that Vorinostat’s protective mechanisms extend beyond those of HIF-1α silencing. Consistently, we found that Vorinostat treatment downregulates HO-1 protein levels under hypoxidative stress, a condition in which HO-1 acts as a prooxidative enzyme. SP2509, a selective inhibitor of lysine-specific demethylase 1 (LSD1), which was reported to protect from ferroptosis by reducing intracellular iron [23], fully rescued ARPE-19 cells from cell death, as well. However, these protective effects were not associated with HIF signaling, because HIF-1α protein levels and HIF-1 transcriptional activity were significantly upregulated by SP2509 treatment. This observation was unexpected, as SP2509 is a selective LSD1 inhibitor and LSD1 was reported to induce HIF-1α stabilization by demethylation [43, 44]. In smooth muscle cells, SP2509 negatively regulated pathways involved in inflammation and ROS production. As such, SP2509 exerted an antioxidative effect at multiple levels by decreasing lipid peroxidation, downregulating HO-1 protein levels, restoring GSH and GSH/GSSG levels, and reducing protein carbonylation. Additionally, it reduced intracellular iron levels by downregulating TFR1 expression under normoxia, suggesting HIF-independent mechanisms [23]. Especially the antioxidative and anti-inflammatory effects of SP2509 are likely to overcome the detrimental effects induced by increased HIF-1 signaling in RPE cells. Thus, SP2509 is another promising approach to protect RPE cells from degeneration in a HIF-independent manner.

In conclusion, the present study highlights the necessity to consider the reciprocal interaction between hypoxia and oxidative stress in the regulation of ferroptosis. Furthermore, our results highlight the potential of targeted HIF inhibition as a potential avenue to improve RPE integrity and protect RPE cells from degeneration. As evidenced by the improvement in RPE cell metabolism resulting from HIF-1α silencing, this approach may serve as a viable strategy for early intervention in age-related visual disorders, such as AMD, preceding the irreversible damage that can be caused to RPE cells and photoreceptors. Furthermore, our data collectively suggest that Vorinostat treatment has strong potential to alleviate ferroptosis and metabolic dysregulation beyond merely suppressing HIF signaling.

## Material and methods

### Cell culture

ARPE-19, an immortalized RPE cell line (Cat. No.: ATCC CRL-2302; Batch No: 70004873), was obtained from American Type Culture Collection (ATCC, Manassas, VA; distributed by LGC Standards GmbH, Wesel, Germany). Cells were authenticated by STR profiling and routinely tested for mycoplasma contamination.

Cells were routinely cultivated in Dulbecco’s modified Eagle’s medium (DMEM)/F-12 with GlutaMAX (31331093, Thermo Fisher Scientific, Waltham, MA) supplemented with 10% fetal bovine serum (FBS) and penicillin–streptomycin (PS) at 37 °C under 21% O_2_ and 5% CO_2_. Upon confluence, FBS was reduced to 1%. All experiments were conducted with DMEM/F12 supplemented with 1% FBS and penicillin–streptomycin within six passages after thawing.

### Hypoxidative stress model

To induce hypoxidative stress, i.e. a combination of hypoxia and oxidative stress, ARPE-19 cells were treated with sodium iodate (NaIO_3_; sc-251029, Santa Cruz Biotechnology, Heidelberg, Germany) under 1% O_2_ (7.5 mmHg) in a hypoxia chamber. NaIO_3_ concentrations between 1-20 mM NaIO_3_ were prepared for the treatments using a freshly prepared 200 mM stock solution in cell culture medium. NaIO_3_ specifically targets RPE cells and resembles key pathological features of geographic atrophy in vitro and in vivo including RPE and photoreceptor degeneration, reduction of electroretinogram (ERG) responses, and complement activation [45–47]. Furthermore, NaIO_3_ leads to the production of superoxide resulting in oxidative damage, similar to lipofuscin, a pigment accumulating with age in the RPE and associated with AMD pathophysiology [48, 49]. Thus, NaIO_3_ is a well-suited oxidative stress agent for preclinical research on RPE degeneration.

### siRNA transfection

For siRNA-mediated knockdown experiments, ARPE-19 cells were seeded at a density of 300,000 cells/well in 6-well plates, 50,000 cells/well in 24-well plates and 10,000 cells/well in 96-well plates in DMEM/F12 with GlutaMAX supplemented with 10% FBS and PS. At approximately 60-70% confluence, cells were transfected with the target siRNA as well as a non-targeting control using Lipofectamine RNAiMAX Transfection Reagent (13778150, Thermo Fisher Scientific, Waltham, MA) and Opti-MEM (31985062, Thermo Fisher Scientific) according to the manufacturer’s protocol. After transfection, cells were cultured for another 48 h before conducting experiments. The following siRNAs were used: ON-TARGETplus Non-targeting Control siRNA (D-001810-01-20, Horizon Discovery, Waterbeach, UK), ON-TARGETplus Human HIF1A siRNA (L-004018-00-0010, Horizon Discovery), Silencer Select Human EPAS1 siRNA (4427038 s4698, Thermo Fisher Scientific), ON-TARGETplus Human TFRC siRNA (L-003941-00-0005, Horizon Discovery), ON-TARGETplus Human HMOX1 siRNA (L-006372-00-0005, Horizon Discovery).

### Inhibitor treatment

Confluent ARPE-19 cells were treated with each inhibitor or vehicle solution under hypoxic (1% O_2_) or hypoxidative stress (10 mM NaIO_3_ at 1% O_2_) conditions. The following inhibitors were used: Ferrostatin-1 (Fer-1; ab146169, Abcam, Cambridge, UK), Deferoxamine Mesylate (ab120727, Abcam), 2’2-Bipyridyl (D216305-2, Sigma Aldrich), Vorinostat/SAHA (S1047, Selleck Chemicals GmbH, Cologne, Germany), SP2509 (S7680, Selleck Chemicals), Belzutifan/PT2977 (S8886, Selleck Chemicals), Bortezomib (S1013, Selleck Chemicals), and IDF-11774 (S8771, Selleck Chemicals).

### C11-BODIPY 581/591 staining

To measure lipid peroxidation, ARPE-19 cells were cultured in black 96-well plates. Treatment with siRNA and inhibitors was conducted as described above and cells were treated for 18 h under hypoxic (1% O_2_) or hypoxidative stress (10 mM NaIO_3_ at 1% O_2_) conditions. Subsequently, cells were washed with PBS and treated with C11-BODIPY 581/591 (Cay27086-1, Biomol, Hamburg, Germany), a fluorescent indicator of lipid peroxidation, at a concentration of 5 µM in serum-free DMEM/F12 for 30 min in the hypoxia chamber. Cells were washed with PBS under hypoxia and fluorescence signal from oxidized and reduced C11-BODIPY 581/591 was measured using a fluorescence plate reader.

### LDH assay

To assess cell viability, ARPE-19 cells were seeded in 96-well plates. Treatment with siRNA or inhibitors was conducted as described above. Confluent cells were exposed to hypoxidative stress (10 mM NaIO_3_ at 1% O_2_) for 24 h. Subsequently, 50 µL cell culture medium was transferred to a 96-well plate and LDH release into medium was measured using a CyQUANT™ LDH Cytotoxicity Assay (C20301, Thermo Fisher Scientific) according to the manufacturer’s instructions. Absorbance was measured at 490 nm and 680 nm (background) with a plate reader and relative LDH release was calculated using maximum LDH activity controls.

### MTT assay

The MTT assay was performed to measure the number of metabolically active cells as a measure of cell viability in HIF knockdown cells. For this purpose, ARPE-19 cells were seeded in 96-well plates at a density of 10,000 cell/well. HIF knockdown was conducted as described above. Upon confluence, cells were exposed to hypoxic (1% O_2_) or hypoxidative stress (10 mM NaIO_3_ at 1% O_2_) conditions for 24 h before adding 50 µL/well (0,5 mg/mL in ddH_2_O) MTT reagent (3-(4,5-dimethylthiazol-2-yl)-2,5-diphenyl-2H-tetrazolium bromide), which is reduced to formazan in metabolically active cells. After 1 h of incubation, the medium was removed and 100 µL DMSO was added to dissolve the formazan crystals. Absorbance was measured at 540 nm and 700 nm (background) with a plate reader. Absorbance was normalized to the corresponding knockdown groups cultivated under hypoxic conditions at 1% O_2_.

### Flow cytometry to determine cell death

To determine cell death by flow cytometry, ARPE-19 cells were seeded in 24-well plates. HIF knockdown was conducted as described above and cells were treated under hypoxic (1% O_2_) or hypoxidative stress (10 mM NaIO_3_ at 1% O_2_) conditions for 24 h. Cells were stained with a Zombie Aqua fixable viability kit (423101, BioLegend, San Diego, CA) according to the manufacturer’s instructions. Data were acquired on a FACSCelesta flow cytometer (BD Biosciences). Data analyses were performed using FlowJo software (BD Biosciences). Cells were first gated based on their size and granularity using a forward scatter versus side scatter plot to exclude debris and aggregates. Subsequently, single cells were identified. The final gating step involved selecting Zombie-positive cells, which indicate compromised membrane integrity and are therefore considered non-viable. An unstained untreated sample was used to establish baseline fluorescence, and a stained killed sample was used to set the Zombie gate.

### Detection of intracellular iron using flow cytometry

Cells were plated in 6-well plates at a density of 300,000 cells/well. HIF-1α and HIF-2α knockdown was conducted as described above and cells were treated under hypoxic (1% O_2_) or hypoxidative stress (10 mM NaIO_3_ at 1% O_2_) conditions for 24 h (24 h treatments consisted of 18 h preincubation at 1% O_2_ followed by 6 h incubation with 10 mM NaIO_3_ at 1% O_2_ to avoid cell death by NaIO_3_). For Fe^3+^ quantification, cell pellets were stained with 10 μM 7-Nitrobenz-2-oxa-1,3-diazole-desferrioxamine (NDB-DFO, Squarix Biotechnology, Marl, Germany) for 12 min at 37 °C as previously described [4]. In brief, NBD-DFO fluorescence is quenched by Fe^3+^ allowing the determination of ferric iron [50]. The mean fluorescence intensity (MFI) was detected by flow cytometry (BD CytoFLEX S, Beckman Coulter; FL-2). Fold changes were quantified relative to the corresponding untreated controls.

### Quantitative real-time PCR

Total RNA was isolated according to the manufacturer’s protocol using the NucleoSpin RNA kit (740955.250, Macherey-Nagel, Düren Germany). 0.5–1 µg total RNA was used to synthesize complementary DNA (cDNA) with M-MLV reverse transcriptase (M1705, Promega, Walldorf, Germany) and oligo-dT primer. Quantitative Real-Time PCR (qRT-PCR) was performed using a Biozym Blue S’Green master mix (331416XL, Biozym Scientific, Hessisch Oldendorf, Germany) on a BioRad CFX Opus 96 System. Relative expression levels were calculated with the ΔΔ*ct* method [51] using hypoxanthine-guanine phosphoribosyltransferase (HPRT) as a reference gene. Primers are listed in Table 1.

**Table 1:** List of primers used for qRT-PCR.

Gene	5’	3’
*HIF1A*	GGATGCTGGTGATTTGGATA	TCATGGTCACATGGATGAGTA
*HIF2A*	CGGAGGTGTTCTATGAGCTGG	AGCTTGTGTGTTCGCAGGAA
*PHD2*	CCAGCTTCCCGTTACAGT	GCACGACACCGGGAAGTT
*PHD3*	TCCTGCTGTTAAGGCTTCCG	CACAGCGAGGGAATGAACCT
*VEGF*	CGAAACCATGAACTTTCTGC	ACAGGACGGCTTGAAGATG
*CA9*	CACGTGGTTCACCTCAGCAC	CAGCGATTTCTTCCAAGCG
*ADM*	AGTCGTGGGAAGAGGGAACT	ATCCGGACTGCTGTCTTCGG
*DMT1*	CTTTGCCAATGGACTAGGCT	CTTCTGTCAGCAGGCCTTTAG
*TFR1*	ACTTCTTCCGTGCTACTTCCAG	ACTCCACTCTCATGACACGATC
*FRPN*	AAAATCCCTGGGCCCCTTTT	TAGTCGGCCAAGGATCCACA
*DCYTB*	TCGGCACTGCTCGTCG	CACGGCCACCACAGAGATAAT
*FTL*	CCATGAGCTCCCAGATTCGT	GGTCGAAATAGAAGCCCAGAGA
*FTH*	TCAACAGTGCTTGGACGGAA	GTCCTGGTGGTAGTTCTGGC
*GPX1*	AGTCGGTGTATGCCTTCTCG	TCTTGGCGTTCTCCTGATGC
*GPX4*	GTGGAAGTGGATGAAGAT	GATGAGGAACTTGGTGAA
*SOD1*	AGGCATGTTGGAGACTTGGG	TGCTTTTTCATGGACCACCAG
*SOD2*	CGTTGGCCAAGGGAGATGTT	CACGTTTGATGGCTTCCAGC
*NRF2*	CCAACTACTCCCAGGTTGCC	AAGTGACTGAAACGTAGCCGA
*NQO1*	CAGTGGTTTGGAGTCCCTGC	ACTGCCTTCTTACTCCGGAAGG
*HO1*	TCCTGGCTCAGCCTCAAATG	CGTTAAACACCTCCCTCCCC
*KEAP1*	CCCAACCGACAACCAAGACC	GCACTCAGGGACCTTGGC
*HPRT*	CCTGGCGTCGTGATTAGTGA	CGAGCAAGACGTTCAGTCCT

### Western blot

Cells were seeded at a density of 300,000 cells/well in a 6-well plate and confluent cells were treated under hypoxic (1% O_2_) or hypoxidative stress (10 mM NaIO_3_ at 1% O_2_) conditions for 6 h or 24 h (24 h treatments consisted of 18 h preincubation at 1% O_2_ followed by 6 h incubation with 10 mM NaIO_3_ at 1% O_2_ to avoid cell death by NaIO_3_). For protein isolation, cells were washed with cold PBS, collected in lysis buffer using a cell scraper and incubated on ice for 20 min. After centrifugation for 5 min at 500 *×g* at 4 °C, the supernatant was stored at −80 °C until use. 30 µg of total protein was incubated in Laemmli sample buffer for 5 min at 95 °C, separated by SDS–PAGE and transferred to a PVDF membrane using the Trans-Blot Turbo Transfer System (Bio-Rad Laboratories, Feldkirchen, Germany). Membranes were blocked with 5% non-fat dry milk in TBS-T for 1 h at room temperature. Primary antibodies and secondary antibodies were diluted in blocking buffer and incubated overnight at 4 °C and 1 h at room temperature, respectively. Signals were developed with SuperSignal West Femto Maximum Sensitivity Substrate (34096, Thermo Fisher Scientific) and detected with a Fusion FX System (Vilber, Eberhardzell, Germany). ImageJ software (ImageJ 1.54 g, NIH, Bethesda, Md.) was used for quantification of protein bands. Uncropped Western blot images are provided in Supplementary File 1.

The following antibodies were used: HIF-1α (610958, BD Biosciences, Franklin Lakes, NJ), HIF-2α (NB100-122, Novus Biologicals, Littleton, CO), TFR1/CD71 (66180-1-IG, Proteintech Group, Rosemont, IL), NRF2 (ab62352, Abcam, Cambridge, UK), HO-1 (43966S, Cell Signaling Technology), α-Tubulin (sc-8035, Santa Cruz Biotechnology, Dallas, TX), as well as goat anti-mouse (A2304, Sigma Aldrich) and goat anti-rabbit (A0545, Sigma Aldrich) secondary antibodies.

### Extracellular Flux Assay – Seahorse Analysis

ARPE-19 cells were plated at a density of 20,000 cells/well in XF24 cell culture microplates (Agilent Technologies) in DMEM/F12 supplemented with 10% FBS and cultivated in a humified incubator at 37 °C with 5% CO_2_. On the next day, siRNA-mediated HIF-1α and HIF-2α knockdown was conducted as described above and cells were cultivated for another 48 h under normoxia prior to the treatments. Confluent cells were treated under hypoxic (1% O_2_) or hypoxidative stress (1 mM NaIO_3_ at 1% O_2_) conditions for 24 h. 1 h prior to the Seahorse assay time point, the medium was exchanged to the Seahorse XF DMEM Medium pH 7.4 (103680-100, Agilent Technologies) supplemented with 1 mM sodium pyruvate, 2 mM glutamine and 10 mM glucose, and incubated at 37 °C without CO_2_ under hypoxia (1% O_2_) prior to metabolic flux measurements under hypoxia. During assays, oxygen consumption rate (OCR) and extracellular acidification rate (ECAR) were measured in parallel using a Seahorse XFe24 Analyzer (Agilent Technologies). For real-time analysis of mitochondrial respiratory capacity, the Mitochondrial Stress Test was conducted using the following injections: oligomycin (1 μM), carbonyl cyanide-4-(trifluoromethoxy) phenylhydrazone (FCCP, 0.5 μM), and a combined injection of rotenone and antimycin A (0.5 μM each). For analysis of glycolytic capacity, Glycolytic Rate Assay was conducted using the following injections: a combined injection of rotenone and antimycin A (0.5 μM each) followed by 50 mM 2-desoxyglucose. Proton efflux rate (PER) was calculated by Wave 2.6.1 software. For individual well normalization, cells were stained with 1 µg/mL Hoechst 33342 (Sigma-Aldrich) solution and relative fluorescence intensity (RFU) was measured after each assay. All metabolic parameters were normalized to Hoechst intensity (RFU) in each well. Data were analyzed using Wave 2.6.1 software (Agilent Technologies).

### Statistics

Statistical analyses were performed using GraphPad Prism (version 9.5.1, San Diego, CA, USA). All data were tested for normal distribution using Anderson–Darling, D’Agostino–Pearson omnibus, and Shapiro–Wilk normality test. Unpaired t-test, as well as one-way ANOVA, two-way ANOVA followed by Tukey’s multiple comparisons test, and mixed-effects model followed by Šídák’s multiple comparisons test were performed on normally distributed data. If datasets were not normally distributed, data were log-transformed and checked for outliers by using residual or QQ plots. If normal distribution was not achieved, data were analyzed using non-parametric tests. Sample sizes were chosen as reported previously [4] and are indicated in the respective figure legends. All data are expressed as mean ± SD or SEM (as indicated). Statistical significance was defined as *p < 0.05, **p < 0.01, ***p < 0.001, and ****p < 0.0001.

## Supplementary information

Supplementary Figures

Original Data

## Data Availability

The datasets generated in the current study will be made available upon request.
