# Supplementary material for: Targeting hypoxia-inducible factor-1 in a hypoxidative stress model protects retinal pigment epithelium cells from cell death and metabolic dysregulation
Source: Cell Death Discov. 2025 Aug 14;11:380. doi: 10.1038/s41420-025-02675-7 (PMC12354819; doi:10.1038/s41420-025-02675-7)
Supplement: Supplementary file 1 — Supplementary Figures [file 41420_2025_2675_MOESM1_ESM.pdf]

Supplementary Figures

Targeting hypoxia-inducible factor-1 in a hypoxidative stress model protects retinal pigment epithelium cells from cell death and metabolic dysregulation

Annika Schubert, Maria Eduarda Lobo Barbosa da Silva, Tabea Ambrock, Orbel Terosian, Anna Malyshkina, Claudia Padberg, Safa Larafa, Johann Matschke, Joachim Fandrey, Yoshiyuki Henning

HIF-associated genes

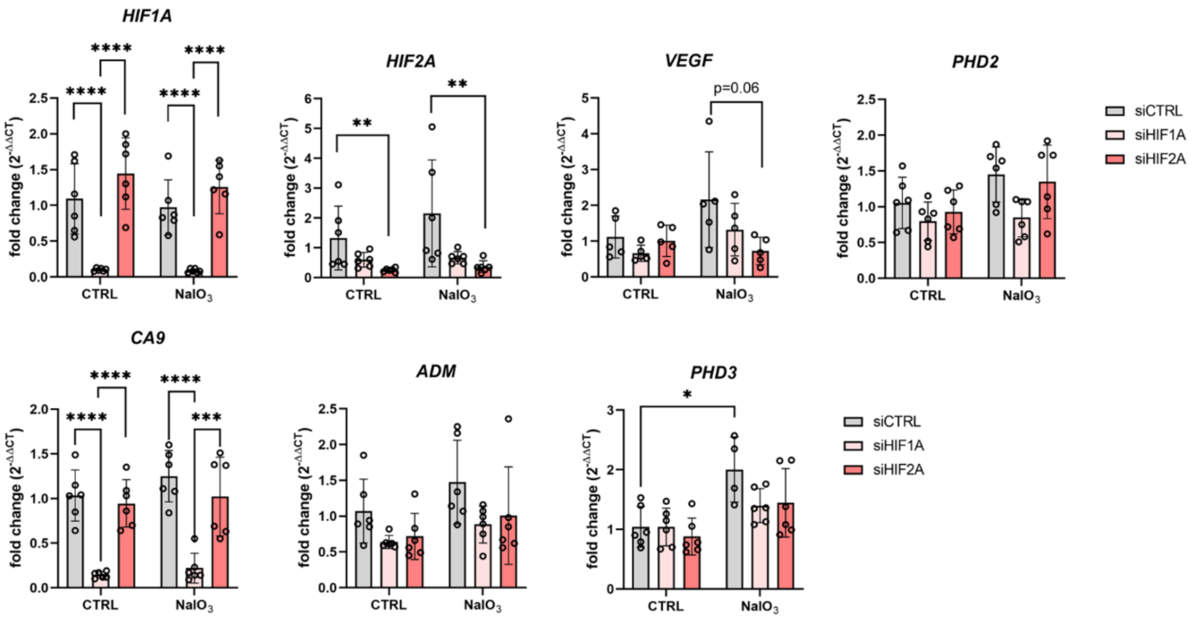

Supplementary Figure S1: Screening of HIF-associated genes. ARPE-19 cells with *HIF1A* and *HIF2A* knockdown were treated under hypoxia or hypoxidative stress conditions to test the effects on the expression of HIF-associated genes by qRT-PCR (N=4–6). Data were statistically analyzed with two-way ANOVA followed by Tukey's multiple comparisons test. All data are expressed as mean ± SD. *****$p < 0.05$, *******$p < 0.001$, and ********$p < 0.0001$.

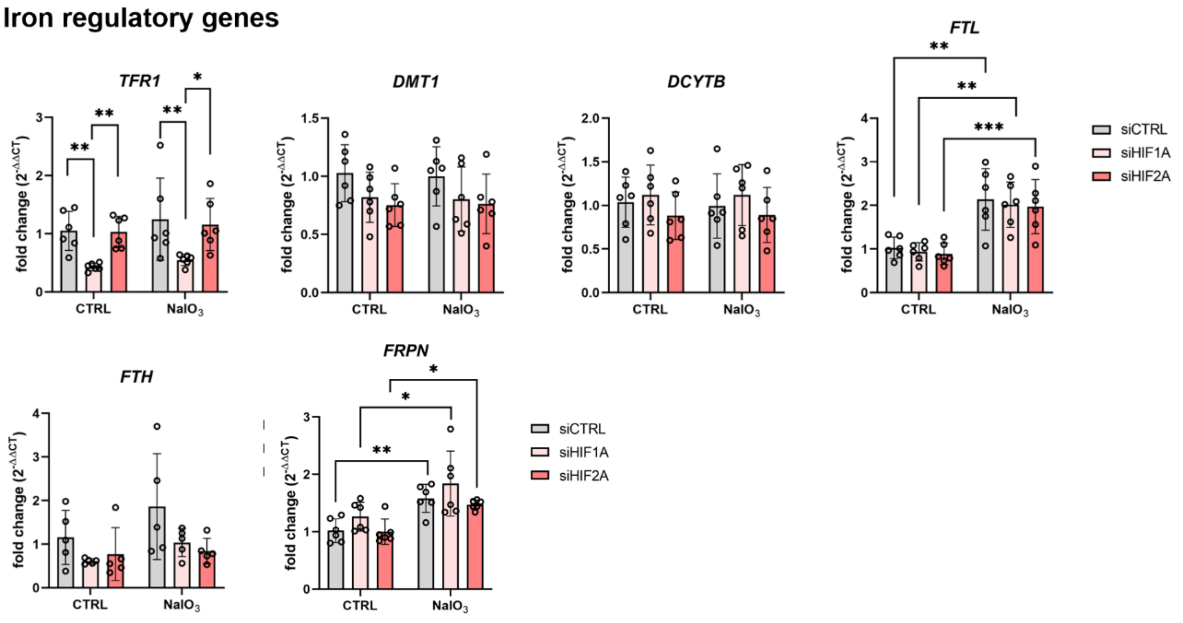

Supplementary Figure S2: Screening of iron regulatory genes. ARPE-19 cells with *HIF1A* and *HIF2A* knockdown were treated under hypoxia or hypoxidative stress conditions to test the effects on the expression of iron regulatory genes by qRT-PCR (N=5–6). Data were statistically analyzed with two-way ANOVA followed by Tukey's multiple comparisons test. All data are expressed as mean ± SD. *****$p < 0.05$, ******$p < 0.01$, and *******$p < 0.001$.

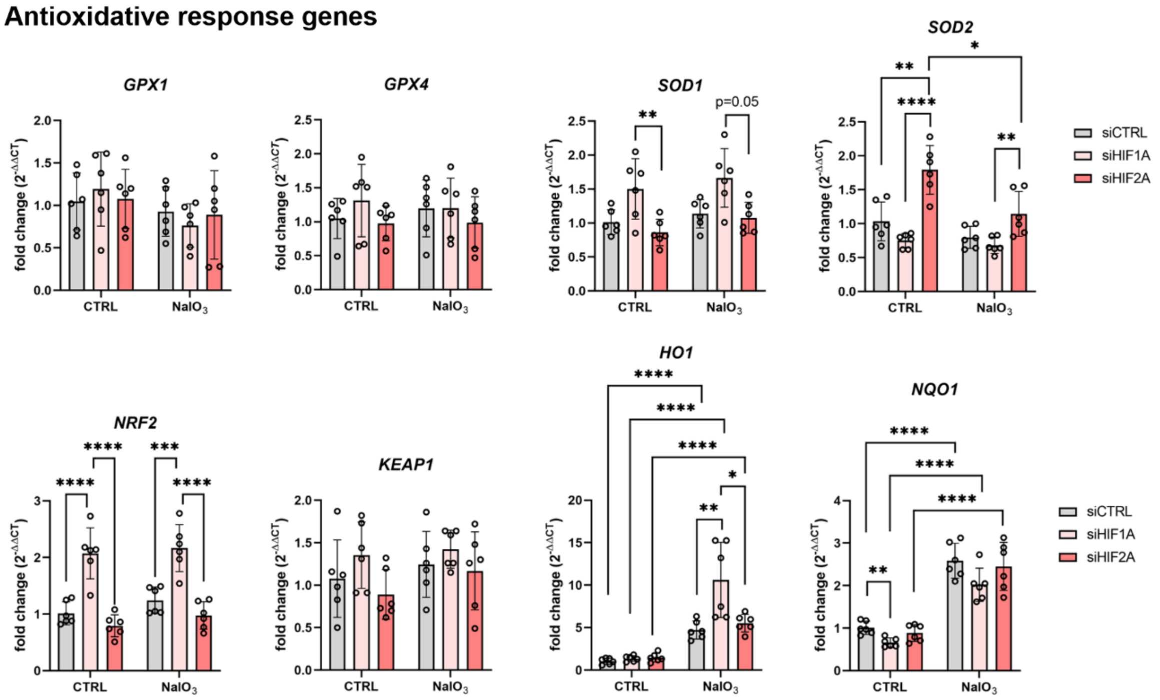

Supplementary Figure S3: Screening of antioxidative response genes. ARPE-19 cells with *HIF1A* and *HIF2A* knockdown were treated under hypoxia or hypoxidative stress conditions to test the effects on the expression of antioxidative response genes by qRT-PCR (N=5–6). Data were statistically analyzed with two-way ANOVA followed by Tukey's multiple comparisons test. All data are expressed as mean ± SD. *****$p < 0.05$, ******$p < 0.01$, *******$p < 0.001$, and ********$p < 0.0001$.

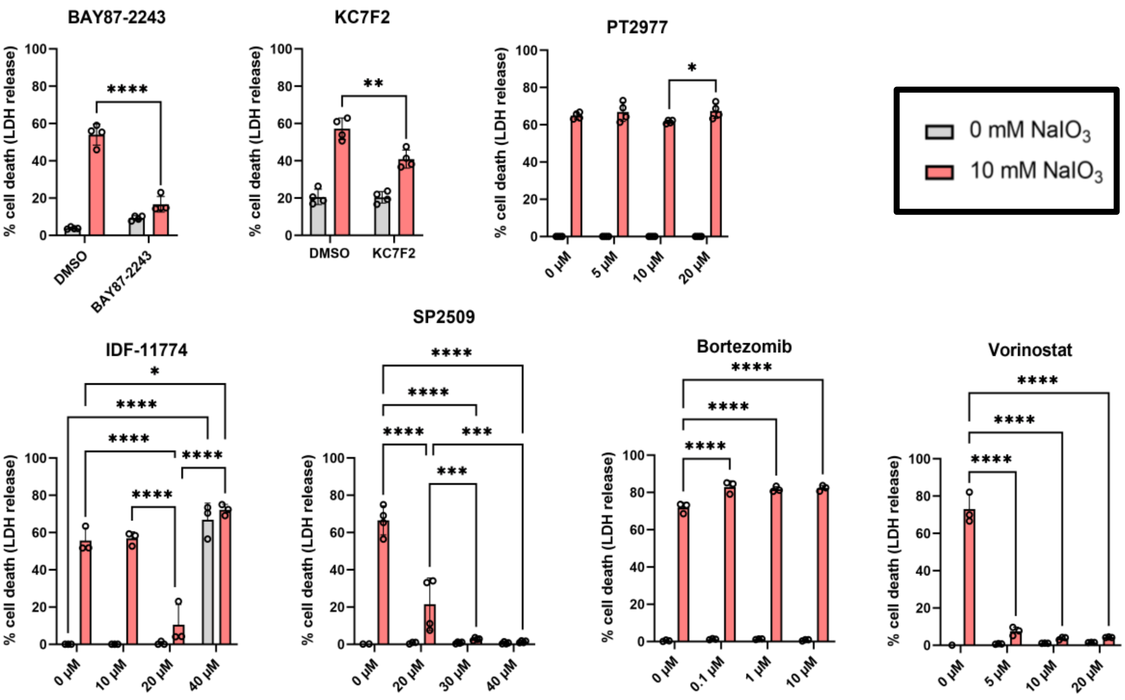

Supplementary Figure S4: Screening of potential HIF inhibitors. ARPE-19 cells were treated under hypoxia or hypoxidative stress conditions for 24h with each small molecule inhibitor or vehicle (DMSO). LDH assays were performed to test the potential of each inhibitor to protect cells from hypoxidative stress-induced cell death (N=3–4). Data were statistically analyzed with two-way ANOVA followed by Tukey's multiple comparisons test. All data are expressed as mean ± SD. *$p < 0.05$, **$p < 0.01$, ***$p < 0.001$, and ****$p < 0.0001$.
