# Supplementary material for: Targeting hypoxia-inducible factor-1 in a hypoxidative stress model protects retinal pigment epithelium cells from cell death and metabolic dysregulation
Source: Cell Death Discov. 2025 Aug 14;11:380. doi: 10.1038/s41420-025-02675-7 (PMC12354819; doi:10.1038/s41420-025-02675-7)

Supplementary File 1

Uncropped Western blot images

Targeting hypoxia-inducible factor-1 in a hypoxidative stress model protects retinal pigment epithelium cells from cell death and metabolic dysregulation

Annika Schubert, Maria Eduarda Lobo Barbosa da Silva, Tabea Ambrock, Orbel Terosian, Anna Malyshkina, Claudia Padberg, Safa Larafa, Johann Matschke, Joachim Fandrey, Yoshiyuki Henning

Fig. 2B.

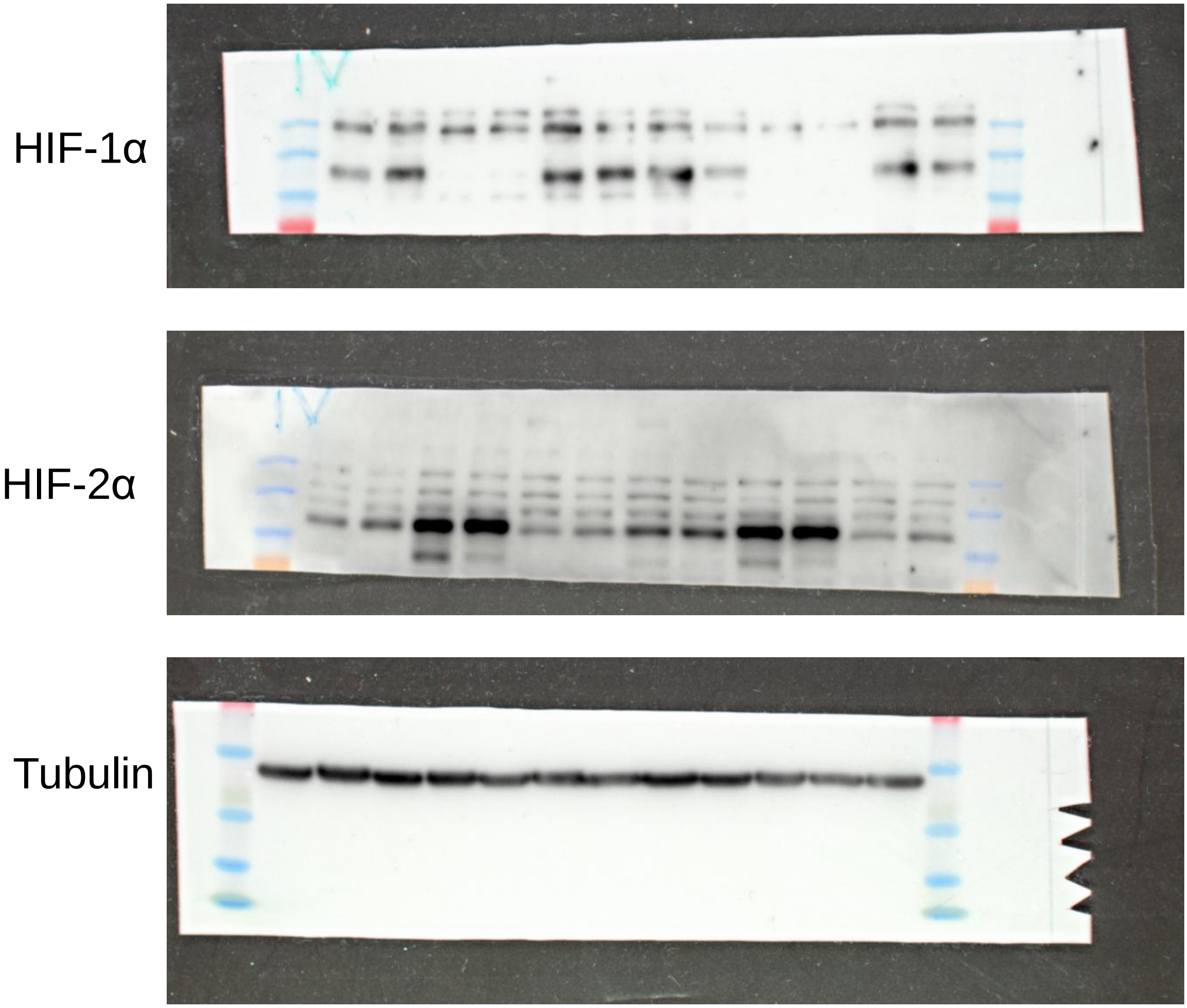

Fig. 2C.

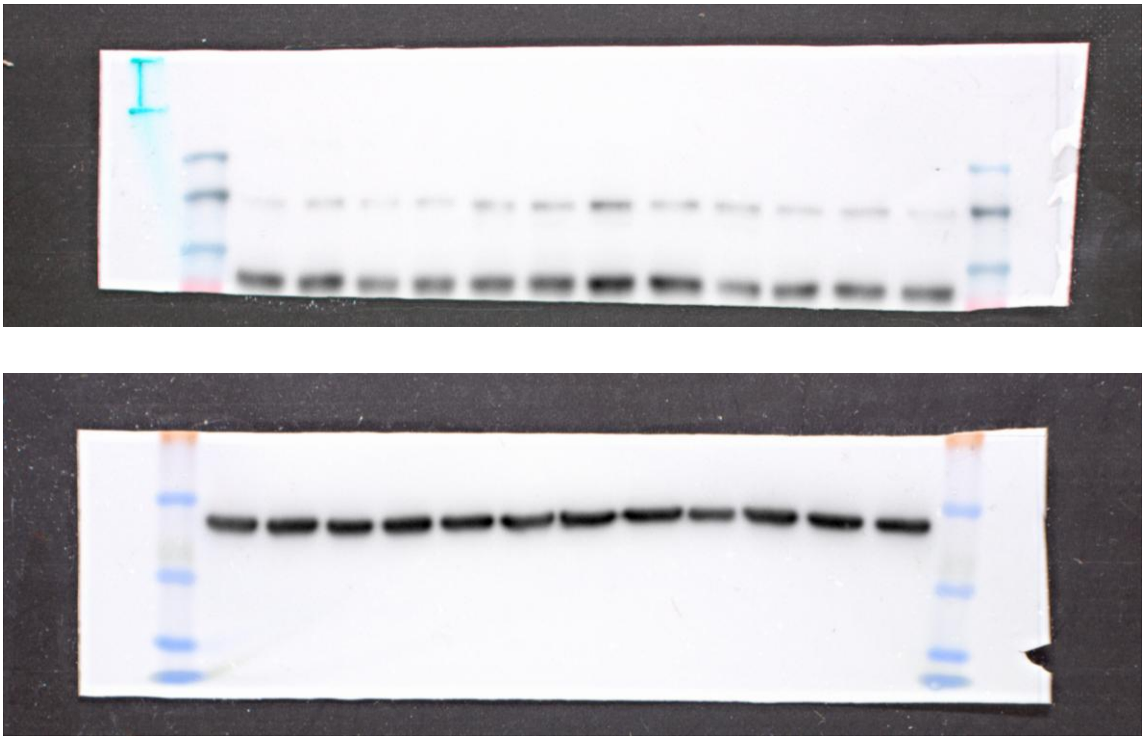

TFR1

Tubulin

Fig. 2D.

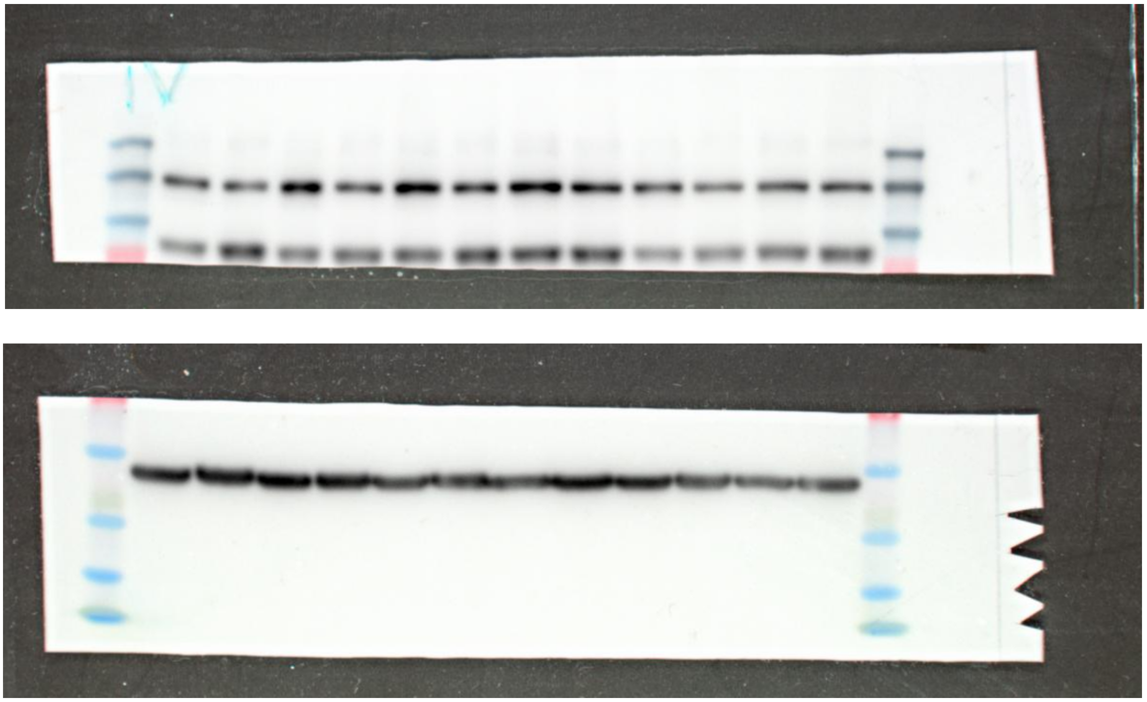

TFR1

Tubulin

Fig. 3B.

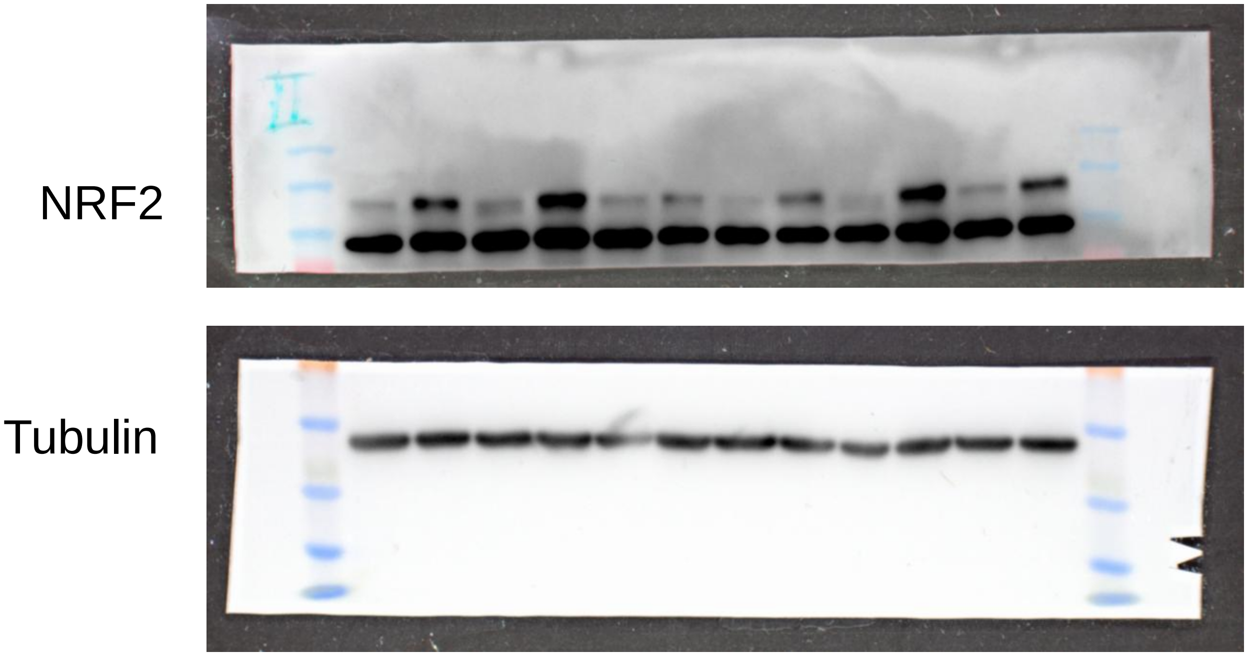

Fig. 3C.

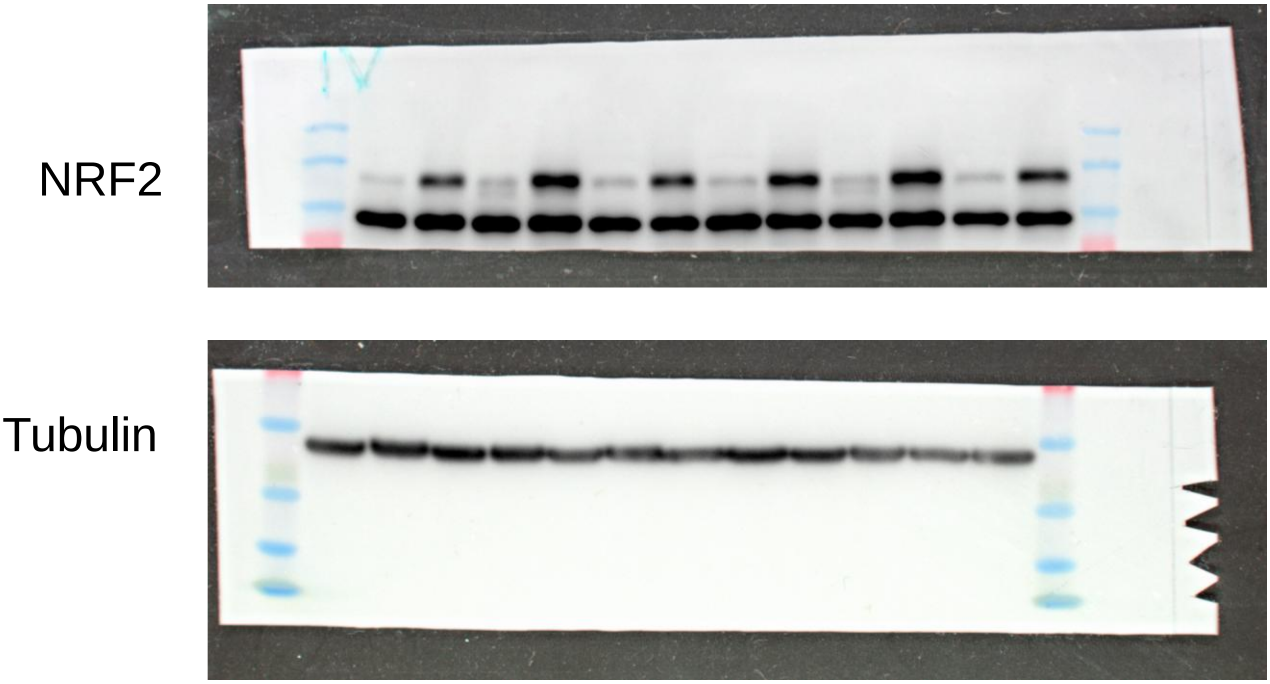

Fig. 3D.

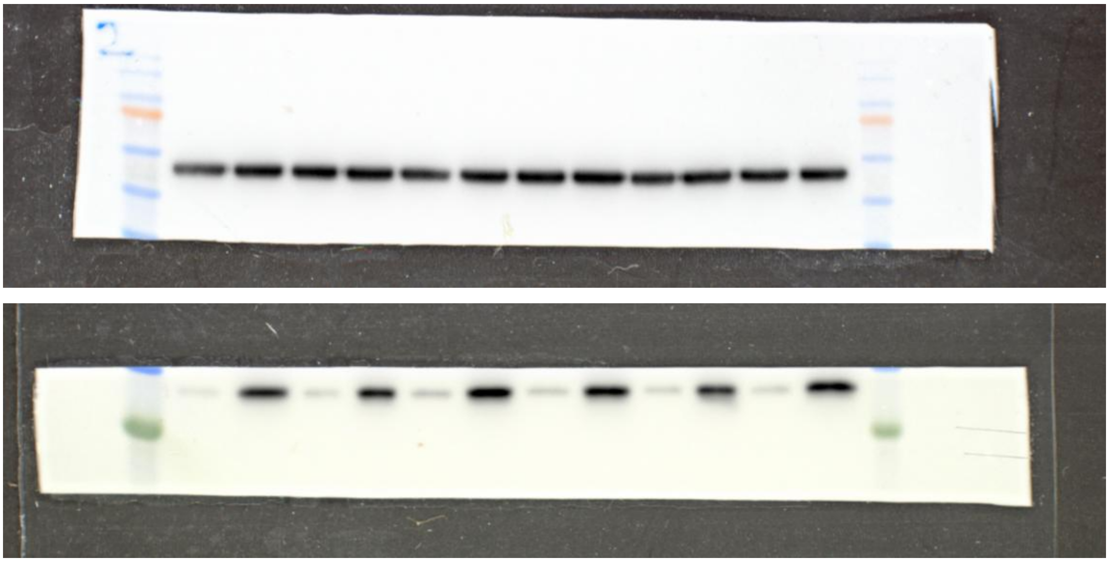

Fig. 3E.

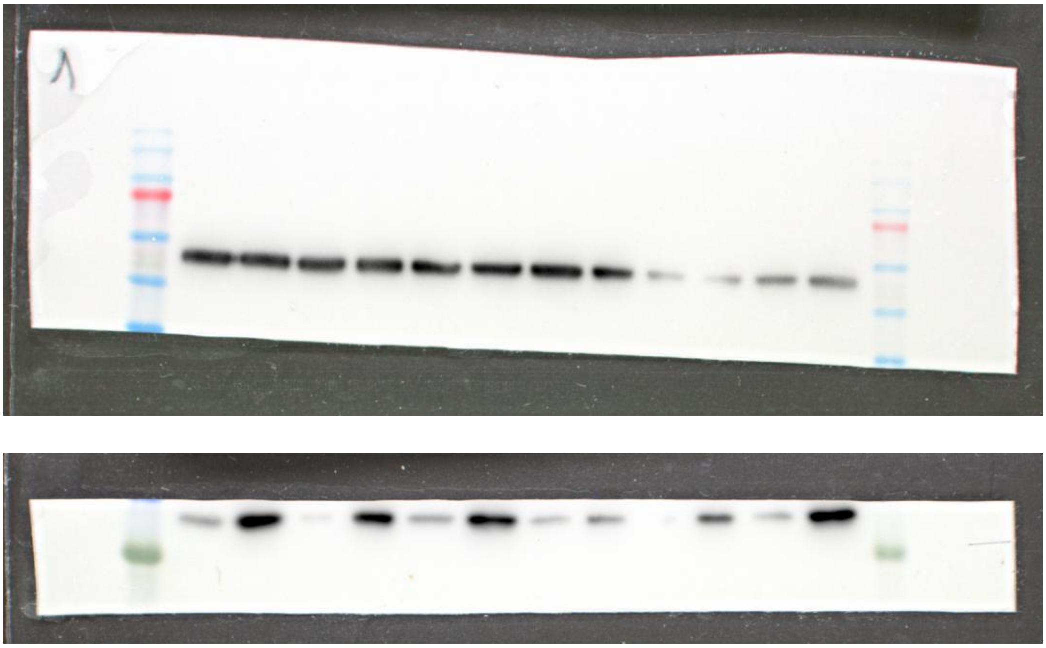

Fig. 5C – SP2509 6h.

HIF-1α

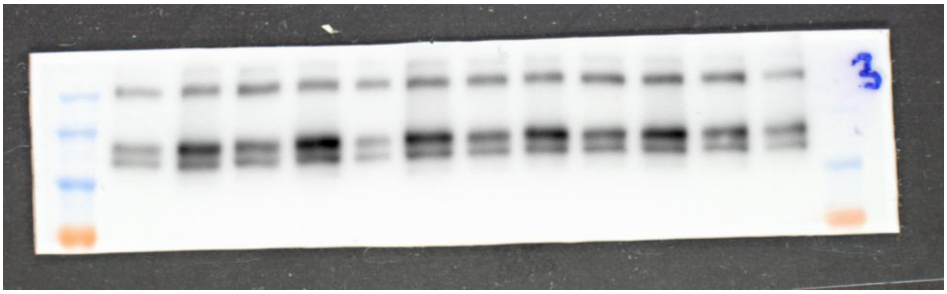

Tubulin

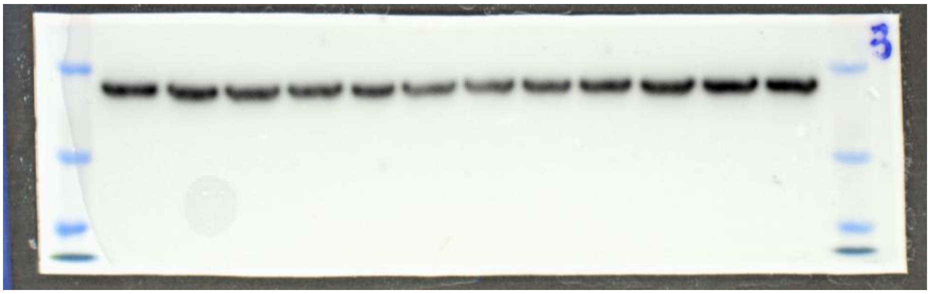

Fig. 5C – Vorinostat 6h.

HIF-1α

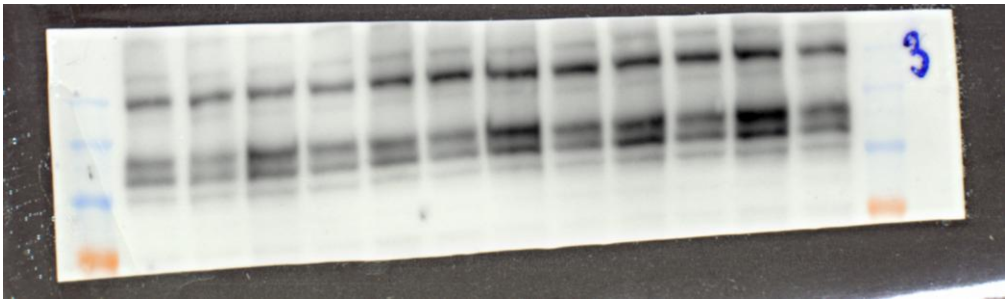

Tubulin

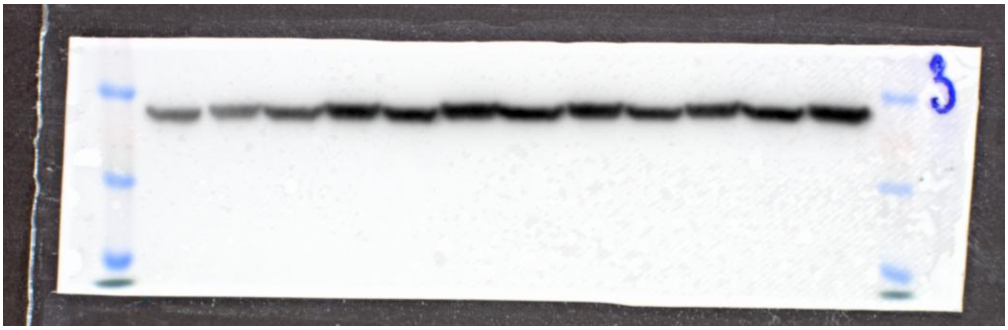

Fig. 5E – Vorinostat 24h.

TFR1

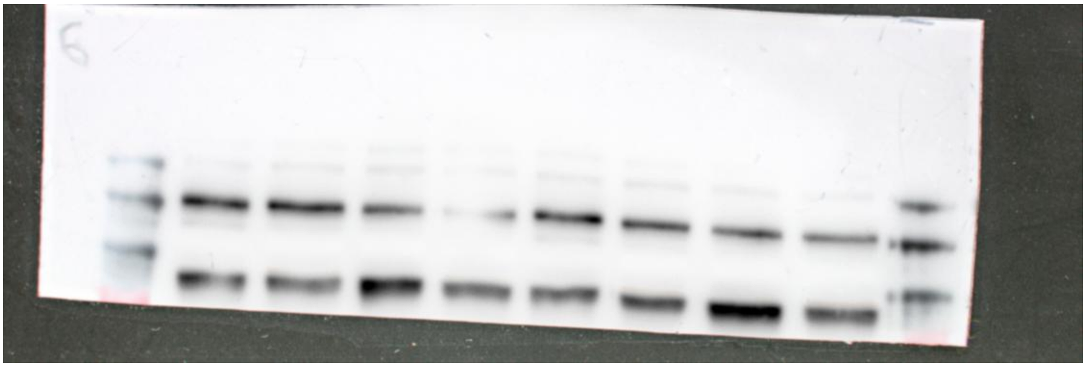

Tubulin

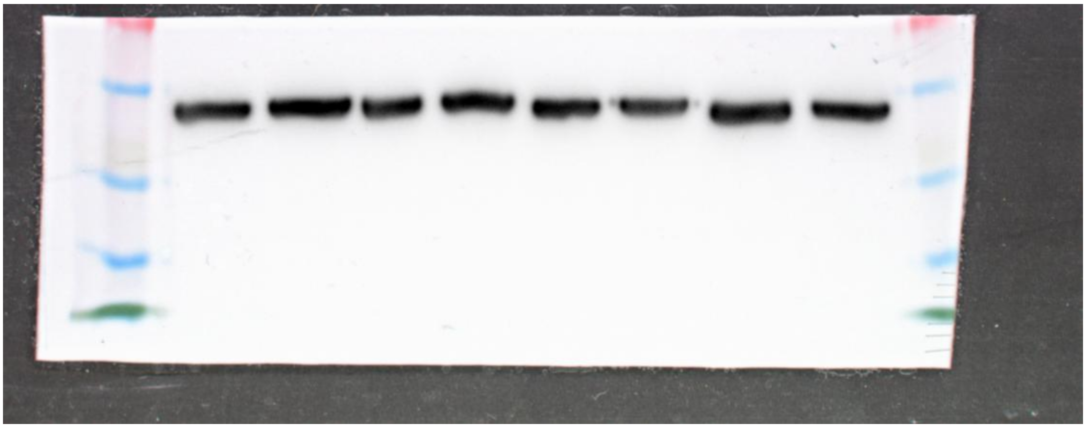

Tubulin

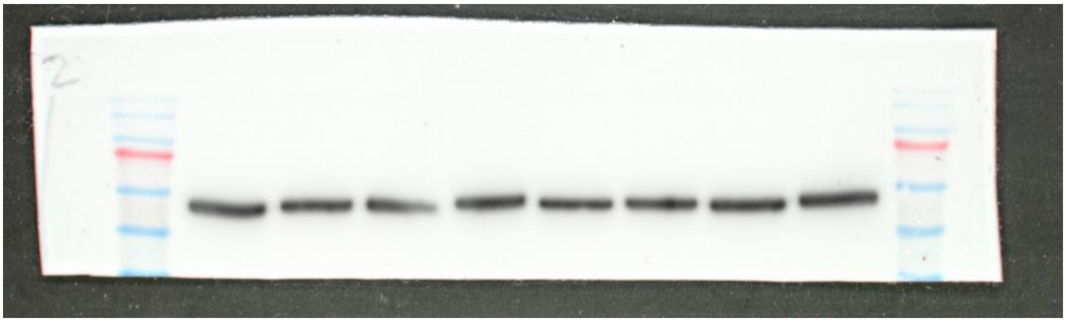

HO-1

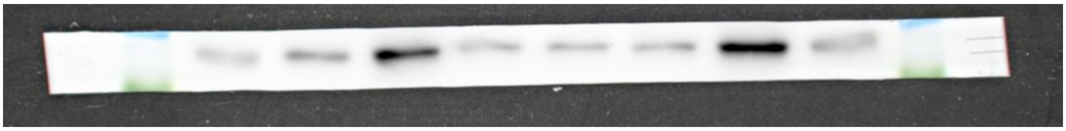

Supplement: Supplementary file 2 — Original Data [file 41420_2025_2675_MOESM2_ESM.pdf]
